# Ribosomopathy-like properties of murine and human cancers

**DOI:** 10.1371/journal.pone.0182705

**Published:** 2017-08-18

**Authors:** Sucheta Kulkarni, James M. Dolezal, Huabo Wang, Laura Jackson, Jie Lu, Brian P. Frodey, Atinuke Dosunmu-Ogunbi, Youjun Li, Marc Fromherz, Audry Kang, Lucas Santana-Santos, Panayiotis V. Benos, Edward V. Prochownik

**Affiliations:** 1 Division of Hematology/Oncology, Children’s Hospital of Pittsburgh of UPMC, Pittsburgh, PA, United States of America; 2 Division of Newborn Medicine, Children’s Hospital of Pittsburgh of UPMC, Pittsburgh, PA, United States of America; 3 College of Life Sciences and The State Key Laboratory of Virology, Wuhan University, Wuhan, Hubei Province, People’s Republic of China; 4 Department of Computational and Systems Biology, The University of Pittsburgh, Pittsburgh, PA, United States of America; 5 Department of Biomedical Informatics, The University of Pittsburgh, Pittsburgh, PA, United States of America; 6 The Department of Microbiology and Molecular Genetics, The University of Pittsburgh School of Medicine, Pittsburgh, PA, United States of America; 7 The University of Pittsburgh Cancer Institute, Pittsburgh PA, United States of America; German Cancer Research Center, GERMANY

## Abstract

Ribosomopathies comprise a heterogeneous group of hematologic and developmental disorders, often characterized by bone marrow failure, skeletal and other developmental abnormalities and cancer predisposition. They are associated with mutations and/or haplo-insufficiencies of ribosomal proteins (RPs) and inefficient ribosomal RNA (rRNA) processing. The resulting ribosomal stress induces the canonical p19^*ARF*^/Mdm2/p53 tumor suppressor pathway leading to proliferative arrest and/or apoptosis. It has been proposed that this pathway is then inactivated during subsequent neoplastic evolution. We show here that two murine models of hepatoblastoma (HB) and hepatocellular carcinoma (HCC) unexpectedly possess features that mimic the ribosomopathies. These include loss of the normal stoichiometry of RP transcripts and proteins and the accumulation of unprocessed rRNA precursors. Silencing of p19^*ARF*^, cytoplasmic sequestration of p53, binding to and inactivation of Mdm2 by free RPs, and up-regulation of the pro-survival protein Bcl-2 may further cooperate to drive tumor growth and survival. Consistent with this notion, re-instatement of constitutive p19^*ARF*^ expression in the HB model completely suppressed tumorigenesis. In >2000 cases of human HCC, colorectal, breast, and prostate cancer, RP transcript deregulation was a frequent finding. In HCC and breast cancer, the severity of this dysregulation was associated with inferior survival. In HCC, the presence of RP gene mutations, some of which were identical to those previously reported in ribosomopathies, were similarly negatively correlated with long-term survival. Taken together, our results indicate that many if not all cancers possess ribosomopathy-like features that may affect their biological behaviors.

## Introduction

The ribosomopathies are clinically heterogeneous disorders characterized by progressive bone marrow failure and a predisposition to hematopoietic and non-hematopoietic malignancies [1, 2]. Most ribosomopathies such as Diamond-Blackfan anemia (DBA), Schwachman-Bodian-Diamond syndrome (SBDS) and dyskeratosis congenital [3] are inherited conditions that are also variably associated with extra-hematopoietic defects including skeletal malformations, exocrine pancreatic insufficiency and growth and cognitive impairment [1, 2]. An acquired ribosomopathy, the 5q- syndrome, is associated with myelofibrosis and evolution to acute myelogenous leukemia in ~10% of cases [3, 4]. Some pediatric T-cell leukemias and certain solid tumors, are also now thought to be acquired ribosomopathies [5]. Although originally considered as distinct clinical entities, the ribosomopathies are related by virtue of their association with hemizygous mutations in ribosomal protein genes leading to RP haplo-insufficiency or malfunction [1]. For example, DBA mutations involve haploinsufficiency or mutation in one of at least 11 of the ~80 RPs comprising the 40S or 60S ribosomal subunits, most notably RPS19, RPS24, RPS26, RPL5 and RPL11 [1, 6]. Similarly, SBDS involves mutations in the SBDS protein, which is believed to promote and/or stabilize the 40S and 60s ribosomal subunit interaction [1, 2]. The minimal chromosomal region deleted in the 5q- abnormality, involving the band 5q33.1, includes the *RPS14* gene [4, 7]. Ribosomopathy-associated bone marrow failure has been attributed to ribosome assembly defects arising as a consequence of altered RP stoichiometry and leading to defective rRNA processing, ribosomal stress and p53-dependent growth arrest and/or apoptosis [1, 3, 4, 8, 9]. Precisely how this predisposes to cancer remains speculative although it presumably involves circumventing or disabling this potent tumor suppressor pathway [10]. Moreover, with the exception of the scattered reports mentioned above, the role of RP transcript and/or protein dysregulation in other human and murine cancers has not been studied.

In the current work, we utilized two mouse models of liver cancer in which the over-expression of mutant forms of β-catenin and yes-associated protein [11] or the oncoprotein c-Myc (Myc), recapitulate pediatric hepatoblastoma (HB) [12] or hepatocellular carcinoma (HCC), respectively [13, 14]. As expected for such rapidly growing tumors, and as previously described for transient Myc over-expression in the liver, virtually all transcripts encoding ribosomal proteins (RPs) were up-regulated in these tumors [15, 16]. However, in comparison to normal hepatocytes or livers, the relative levels of expression for many RP transcripts were deregulated. This was accompanied by distinct alterations in the RP expression patterns of each tumor type, by the accumulation of rRNA precursors and by several defects in the p19^*ARF*^/Mdm2/p53 pathway, most notably a marked reduction in p19^*ARF*^ expression. The reinstatement of p19^*ARF*^ expression completely abrogated *de novo* HB tumorigenesis thereby mechanistically linking this tumor suppressor pathway with those regulated by β-catenin and YAP. In four human cancer cohorts from The Cancer Genome Atlas (TCGA), comprising 2260 individuals, RP transcript deregulation was observed in virtually all tumors when compared to their corresponding normal tissues. Moreover, 5.2%–14.3% of these cancer cases contained mutations in one or more RPs. Breast cancers and HCCs with the greatest degree of RP transcript deregulation and HCCs with RP mutations also demonstrated an overall inferior long-term survival. Thus, even more so than in “classical” ribosomopathies, experimental murine and naturally occurring human cancers demonstrate profound dysregulation of multiple RP transcripts that undoubtedly evoke ribosomal stress and may impact disease pathogenesis and survival.

## Materials and methods

### Animals

Animal studies were conducted in accordance with the Public Health Service Policy on Humane Care and Use of Laboratory Animal Research (ILAR) Guide for Care and Use of Laboratory Animals. Studies were approved by the Institutional Animal Care and Use Committee (IACUC) at the University of Pittsburgh (Protocol no. 14104886) and were in accord with their published guidelines (http://www.iacuc.pitt.edu/policies). All mice were maintained in microisolator cages at no more the five animals/cage as previously described [17]. They were fed *ad libitum* with standard rodent chow and maintained on 12 hr day-night cycles with appropriate environmental enrichment.

C57BL6 *myc*^*fl/fl*^ (WT) mice and hepatocyte-specific *myc-/-* (KO) mice were genotyped as previously described [17]. HBs were induced in 6–8 wk old WT and KO mice by hydrodynamic tail vein injection (HDTVI) of 10 μg each of Sleeping Beauty (SB) vectors encoding the ΔN90 mutant of β-catenin, the S27A mutant of yes-associated protein [11] and 2 μg of a vector encoding Sleeping Beauty transposase [14, 18]. A SB vector encoding murine p19^*ARF*^ (*Cdk2na*) was generated using approaches similar to those previously described for generating β-catenin and YAP vectors [14, 18]. Following HDTVI, animals were monitored thrice weekly for tumor growth. Criteria for euthanasia were in accord with those published both by The University of Pittsburgh IACUC Tumor Burden Guidelines (Neoplasia Proposals in Rodents) and The National Cancer Institute (https://ncifrederick.cancergov/lasp/acuc/frederick/Media/Documents/ACUC14.pdf). Mice were sacrificed using CO_2_ inhalation followed by cervical dislocation when tumors reached ≤2 cm in diameter, which was well before the size that typically required mandatory euthanasia [16]. None of the tumor-bearing mice showed evidence of distress prior to the end of the study.

HCCs were induced in Tet-O-MYC transgenic mice crossed with LAP-tTA mice as previously described [13, 19]. Mouse strains were obtained from Jackson Labs (Bar Harbor, ME). Myc expression was maintained in an off state by adding doxycycline (100 μg/ml) to the drinking water whereas its removal led to the expression of Myc. The criteria for sacrifice were identical to those described above for HBs. Control livers were obtained from age-matched sibling mice lacking the doxycycline-regulatable *myc* gene and also lacking doxycycline in their drinking water.

### RNA-seq analysis

RNA-seq acquisition using an Illumina NextSeq 500 sequencer (San Diego, CA) and data analyses have been previously described [16, 17]. 85 ribosomal proteins for this analysis were identified as described in S1 File. For HBs, the total number of reads for these 85 RP transcripts within each group of samples (four sets of WT hepatocytes, five sets of KO hepatocytes and five sets each of WT and KO tumors) was arbitrarily set at 100%. The average percent contribution of each RP transcript to the total RP transcript pool in each of the groups was then calculated and displayed as a heat map. Replicates within the WT hepatocyte and WT tumor groups were compared using two-sided t-tests assuming unequal variance to determine which RP transcripts were significantly dysregulated in tumors compared to normal hepatocytes. P-values were adjusted assuming a false discovery rate (FDR) of 5% and transcripts with resulting q-values less than the FDR were designated on the heat map with an asterisk (*). The same procedure was performed for Myc-KO hepatocyte and HB groups, with significant differences designated by a caret (^). Bar graphs summarizing relative percent expression of each RP transcript were generated. Transcripts with significantly different relative expression between WT hepatocytes and HBs were compared to those which differed between Myc-KO hepatocytes and HBs. Those differing in both comparisons were noted to have either the same or opposite directionality, with a summary of these differences compiled into a Venn diagram.

A similar analysis was performed for 82 RP genes in the seven HCC groups (five samples per group), with RPs selected as described in S1 File. These groups included normal livers, livers after 3 days and 7 days of Myc induction (3D and 7D), initial tumors, 3 days and 7 days of tumor regression (3R and 7R), and recurrent tumors [12] that were re-induced following a 2–3 month period of regression of initial tumors. As with HBs, the percent relative expression of each transcript within its own group was calculated, and a heat map was generated, with the relative abundance of RP transcripts in each group listed in the same order as those in control liver. Significant differences in RP transcript expression among the groups were determined as described in the HB analysis. Transcripts with significant differences between tumor and liver (q-value < 0.05) were marked with an asterisk (*), and significant differences between RT and liver were marked with a caret (^). As with the HBs, bar graphs were generated for each of the groups, with error bars indicating one standard deviation and asterisks designating significance.

### Human tumor data

RNA-seq expression results for 77 RP transcripts and clinical data were obtained from The Cancer Genome Atlas (TCGA) for the four cancers of interest: HCC, colorectal adenocarcinoma (CRC), breast adenocarcinoma (BC), and prostate adenocarcinoma (PC), described in further detail in the S1 File. For HCC, there were a total of 50 matched tumors and normal tissues samples with 323 unmatched additional tumors. For CRC there were 41 matched samples and 247 additional unmatched tumors. For BC, there were 113 matched samples and 989 additional unmatched tumors. For PC, there were 52 matched samples and 445 additional unmatched tumors.

### TCGA: Matched sample analysis

For each cancer, RP transcript levels of both normal tissue and tumor were obtained from the RNA-seq data of matched patients. RP transcript expression data was converted into relative percent expression as above, analyzing tumors and normal tissues separately. Variation from the mean for the expression of each RP transcript was explored for individual cancers by first examining the natural variability in relative RP transcript expression in the matched normal samples. For each RP transcript in each matched normal sample, variation from the mean was calculated and expressed in a 3D area map, with different samples listed across the x-axis, RP transcripts listed across the y-axis, and variation in relative expression from the mean relative expression in normal tissues on the y-axis. For most RP transcripts, these differences were ±5–20% across most patients, although the transcripts with the lowest expression (e.g. *Rps27*, *Rpl36A*, *Rpl21*) tended to have greater variability between patients, averaging ±100–300% difference compared to the average.

Variation in relative RP transcript expression for tumors in each cancer cohort was examined in the same manner, by calculating percent difference in relative expression for each RP among the tumors compared to average relative RP expression among the normal tissue samples. Unlike the normal tissues, RP transcript expression varied considerably among patients, with differences averaging ±20–100% for most transcripts. As noted above for normal tissues, transcripts with the lowest relative expression again demonstrated the greatest variance.

To determine if the variation in RP transcript relative expression was significantly greater in tumors than in matched normal tissue, an F-test was performed for each RP transcript comparing tumor samples to normal matched tissues, with the resulting P-values adjusted for an FDR of 5%. 69 of the 77 RP transcripts (90%) reached significance for HCC, 48 of 77 (62%) were significant in CRC, 71 of 77 (92%) were significant for BC and 23 of 77 (30%) significant in PC. Transcripts with the lowest expression in both tumors and normal tissue were also those with the greatest variation, and the F-tests comparing variation in these transcripts between tumors and normal tissue did not reach significance. In order to scale the graphs to better evaluate differences in the other transcripts which did reach significance for their F-tests, these transcripts–*Rps26*, *Rpl9*, *Rps27*, *Rps28*, and *Rpl21*– were excluded from indicated 3D area plots.

### TCGA: Unmatched sample analysis

RP transcript expression levels in unmatched tumor samples were compared to the previously analyzed matched normal tissue samples. P-values for each RP transcript were calculated to reflect the probability of observing a relative expression level that extreme (or more extreme) when compared to the distribution of relative expressions seen in normal tissue. Since relative expression levels for RP transcripts among normal tissue samples were normally distributed, these P-values were calculated as $2X1\text{-}\Phi \left(|\frac{x_{t}\text{-}\mu_{n}}{\sigma_{n}}|\right)$, with x_t_ indicating the relative expression of a specific RP transcript in the sample being examined, and μ_n_ and σ_n_ representing the mean and standard deviation of the relative expression for that RP transcript across normal tissues, respectively. The average number of RP transcripts identified as being significantly deregulated by this method (P < 0.05) in a given cohort (an average of 28.3 per tumor in HCCs, 26.2 in CRCs, 23.6 in BCs and 14.6 in PCs) was compared against a binomial distribution of *B (*77, 0.05) to control for false discovery. The P-values for observing as many significantly deregulated transcripts as we did in each cohort were calculated using this binomial distribution: P = 2.6x10^-17^ for HCCs, P = 2.8x10^-15^ for CRCs, P = 2.1x10^-12^ in BC, and P = 2.6x10^-5^ in PCs.

Total RP transcript “deregulation” in each tumor sample was then calculated as the sum of the absolute value of the differences in relative expression of each RP transcript and the mean RP transcript relative expression across the normal tissue samples. Survival curves comparing patients with tumors in the upper quartile of RP dysregulation to patients with tumors in the lower quartile were generated as described in S1 File. Significance was determined by a log-rank test P < 0.05. Survival differences were significant between upper and lower quartile groups in HCC (P = 0.0435) and BC (P = 0.0046).

### TCGA: Mutation analysis

After accounting for SNPs, mutations for each of the TCGA cohorts were investigated using cBioPortal (http://www.cbioportal.org/) as described in S1 File. Tumor samples in each cohort were then separated into two groups: those with any mutation in any RP and those with no identifiable mutations. Survival curves for the cohorts were generated as described above, with Log-rank test p-value cutoffs set at 0.05. Survival differences were significant only in HCC (P = 0.0006).

### Quantification of rRNA processing

To assess rRNA processing intermediates, we quantified 18S-ITS1, ITS1-5.8S, 5.8S-ITS2 and ITS2-18S junctions using a Power SYBR® Green RNA-to-CT™ 1-Step Kit (Thermo-Fisher) on a StepOnePlus™ Real-Time PCR System (Thermo-Fisher) as described in S1 File with P-values determined using Welch’s t-test.

### Immuno-blotting

Total tissue lysates were prepared in SDS-PAGE lysis buffer (150 mM NaCl; 100 mM Tris-HCl, pH 8.0; 12.5 mM EDTA; 2% SDS; 1% Triton-X100; 0.5% Nonidet-P40; 10% glycerol) containing Complete protease inhibitor cocktail (Sigma-Aldrich) and phosphatase inhibitor cocktail II (Boston BioProducts, Inc., Ashland, MA) that were added to the lysis buffer in the amounts recommended by the suppliers. Samples were further disrupted with a Model 505 sonic dismembrator (setting 3 for 15 sec) (Thermo Fisher). Protein concentrations were then determined with a Pierce BCA Protein Assay Kit (Thermo Fisher). Depending on the proteins under study, 10–50 μg of lysate were used for SDS-PAGE and western blotting to PVDF membranes (Thermo Fisher, Inc.) as previously described [17]. All membranes were blocked for at least two hr. at 4°C in PBS containing 0.1% Tween (PBS-T) and 5% non-fat dry milk. Antibodies, vendors and conditions used for immuno-blotting are shown in Table A in S1 File. Most antibodies were incubated with membranes overnight. Following exhaustive washing in PBS-T, blots were then developed using a Pierce ECL Plus reagent (Thermo Fisher, Inc.) according to the directions provided by the supplier.

### Tissue fractionation and immuno-precipitation

Cytoplasmic and nuclear fractions of liver and tumor tissues were isolated with a NE-PER kit as recommended by the supplier (Thermo Scientific, Rockford, IL, USA). See S1 File for the details of fractionation and immuno-precipitation protocol.

### Mass spectrometry

Regions of interest from silver-stained gels were excised and digested with trypsin as previously described [20]. Excised gel pieces were processed as described in detail in S1 File.

## Results

### Deregulation of RP transcripts and ribosomal RNAs in liver cancer models

**Fig 1:**
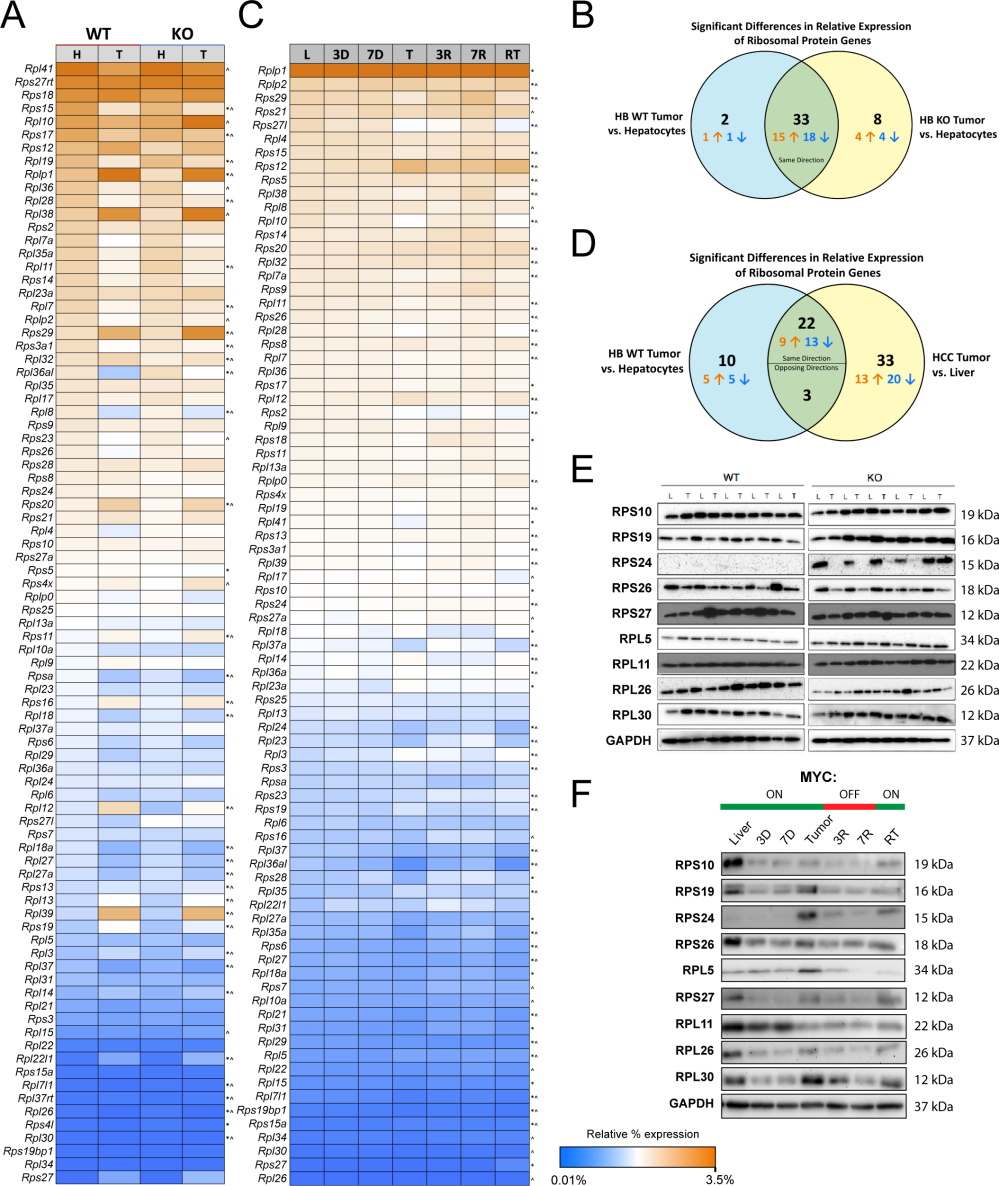
Relative RP transcript and protein levels differ in murine models of HB and HCC. (A) RP transcript abundance in hepatocytes (H) versus HB tumors (T). Heat maps are based on averaged RNA-seq data from 4–5 samples in each group with the most abundant transcripts being shown in orange and the least abundant transcripts being shown in blue, with the sum of all transcripts in each group equaling 100%. All transcript levels are expressed as a percent of total, displayed relative to those in WT hepatocytes and do not take into account the fact, as previously shown, that average RP transcript expression was increased 5.2-fold in HBs relative to hepatocytes [16] *: deregulated transcripts in WT tumors vs. WT hepatocytes, ^: deregulated transcripts in KO tumors vs. KO hepatocytes. (B) RP transcript deregulation among WT and KO hepatocytes and HBs. (C) Similar heat maps from livers or HCCs [13]. L: control livers. 3D and 7D: livers obtained 3 and 7 days after removing doxycycline to induce Myc expression. T: initial tumors. 3R and 7R: regressing tumors following doxycycline resumption for 3 or 7 days, respectively. Additional tumor-bearing mice were maintained on doxycycline for 2.5–3 months to allow for complete regression. Doxycycline removal in these mice led to development of recurrent tumors [12]. *: significant differences in relative expression compared to normal liver; ^: significant differences between recurrent tumor and liver (q-value < 0.05). Relative transcript abundance was expressed as described for panel A and compared with the relative abundance in control livers. (D) RP transcript discordances between HBs and HCCs. “Opposing” directionality occurred when an HB transcript’s direction of change relative to hepatocytes differed between WT and KO HBs. (E) Immunoblots of RPs in WT and KO livers (L) and HB tumors (T). (F) Immunoblots of RPs from livers, collected as described in (C).

In a murine model of HB [12] induced by the over-expression of SB vectors encoding mutant forms of β-catenin and yes-associated protein [11, 14], we recently showed that RP transcripts were, on average up-regulated more than five-fold relative to control, wild-type (WT) hepatocytes [16]. When these results were re-displayed to depict each RP transcript’s percent contribution to the entire RP transcript pool relative to that of untransformed hepatocytes, 35 transcripts from HBs showed significant variation in their abundance (Fig 1A and Figure A in S1 File, q-value <0.05). Prominent examples included *Rpl8* and *Rpl18a*, which were under-expressed in HBs relative to that seen in WT hepatocytes and *Rpl38* and *Rpl39*, which were relatively over-expressed. However, it should again be emphasized that, in absolute terms, all RP transcripts in HBs were up-regulated [16]. HBs arising in livers bearing a hepatocyte-specific deletion of the *myc* gene (KO HBs) and growing more slowly than WT HBs [16] demonstrated a lower degree of absolute RP transcript induction compared to WT HBs (3.6-fold) [16] but nonetheless also showed a similar and highly overlapping RP transcript discordancy (Fig 1A and 1B and Figure B in S1 File). Thus, despite RP transcripts as a group being more highly up-regulated in WT HBs than in KO HBs and the significantly faster growth rates of WT tumors [16], RP transcript discordancy within the two groups was quite similar (Fig 1B) indicating that they appeared to be more related to the transformed state *per se* rather than to the expression of Myc or tumor growth rate.

HCCs induced by the hepatocyte-specific, doxycycline-regulated over-expression of Myc also significantly up-regulate virtually all RP transcripts with a median up-regulation of 3.4-fold [19]. As with HBs (Fig 1A), when the percent contribution of each transcript to the entire RP transcript pool was arranged from highest to lowest and compared to expression pattern in normal control livers, discordancy of 58 RP transcripts was seen in HCCs (Fig 1C and Figure B in S1 File) [13] with 25 of these also being deregulated in HBs (Fig 1D). Importantly, only minimal deregulation of RP transcripts occurred in livers following brief (3 day or 7 day) induction of Myc, which was well before HCC tumors developed. HCC RP transcripts began to normalize within 3 days of Myc silencing, but 56 transcripts were again deregulated in recurrent tumors following 2–3 months of initial tumor regression (Fig 1B and Figure B in S1 File). Thus, maximal RP transcript deregulation appears to require both the continuous over-expression of growth-promoting stimuli such as β-catenin + YAP in HBs or Myc in HCCs and, at least in the latter case, a tumor environment.

We also examined by immuno-blotting the expression of several RPs, the choice of which was dictated by a combination of previously described associations with ribosomopathies and/or the notable dysregulation of their transcripts (Fig 1A and 1C) [1, 2, 4, 7, 10]. At least two of these RPs, RPS24 and RPS26, were expressed at lower levels in most HBs than in livers, particularly in the case of KO tumors (Fig 1E). KO livers also expressed higher levels of RPS24 than WT livers. Several additional RPs, notably RPS19 and RPS27, were sporadically over- or under-expressed in WT and/or KO tumors. Thus, in addition to RP transcripts, multiple RPs also appear to be deregulated. In comparison to HCCs where Myc is the inciting oncogenic insult, Myc’s dispensability for RP transcript discordancy in HBs may at least partly reflect the Myc-independent pathways of β-catenin and YAP signaling that also govern RP transcript expression [16].

HCCs also showed a loss of coordinated RP expression albeit with a pattern distinct from that seen with HBs. Most RPs, such as RPS24, RPS27 and RPL30, were up-regulated in initial tumors whereas RPS19, RPS26 and RPL11 either remained unchanged or even decreased in HCCs (Fig 1F and Figure C in S1 File). Moreover, discordancy between *Rps24* and *Rps27* transcripts were seen in some initial and recurrent tumors. Finally, the kinetics of appearance and disappearance of individual proteins varied considerably. Changes in the levels of several RPs such as RPS10, RPS19 and RPL30 were detectable as early as 3 days after Myc induction and well before deregulation of their transcripts could be detected. Thus, similar to RP transcripts, individual RP protein levels in HCCs do not change uniformly or coordinately during the course of tumor evolution, regression and recurrence with respect to one another. However, the overall pattern of these changes does appear to be consistent among individual tumors and indicates a marked change in RP stoichiometries relative to those found in normal liver.

**Fig 2:**
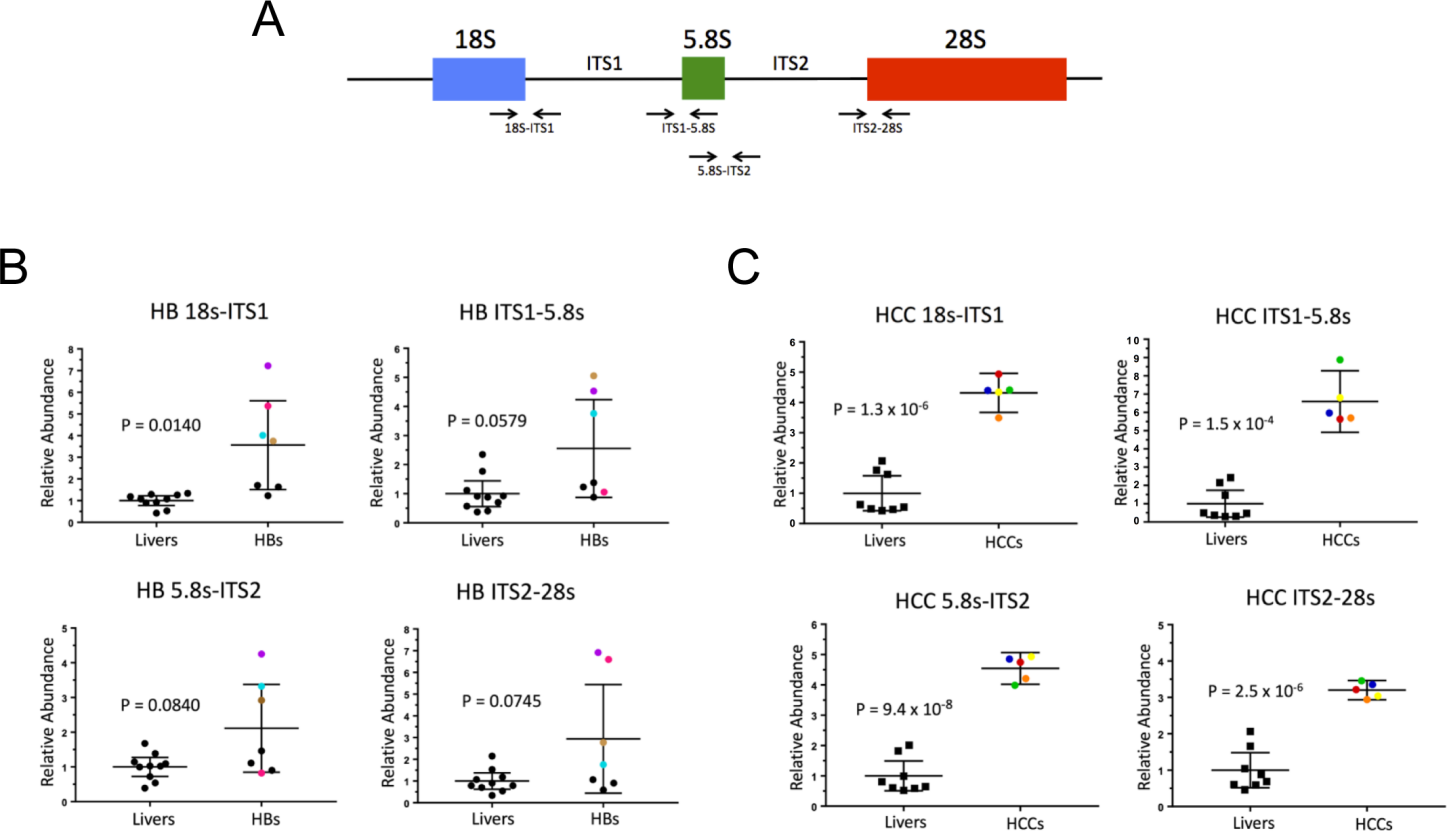
Incomplete processing of rRNAs in HBs and HCCs. (A) Normal rRNA processing. Arrows depict regions amplified by qRT-PCR to quantify 18S-ITS1, ITS1-5.8S, 5.8S-ITS2 and ITS2-28S junctional fragments common to all rRNA precursors. (B) Quantification of each of the above four junctions in control livers and HBs. Identically colored dots represent the same tumor RNA sample within the subgroup of tumors that demonstrated abnormal processing of at least one junction. Control livers and tumors with no significant processing differences are depicted in black. (C) Similar quantification of RNA processing in HCCs. Data in (B) and (C) were normalized to levels of total 18S and 28S RNA. Each qRT-PCR reaction was performed in triplicate and the mean is depicted.

Efficient ribosomal biogenesis requires the highly coordinated processing and assembly of rRNAs as well as RPs [21, 22]. 18S, 5.8S and 28S rRNA are transcribed from a single primary transcript 47S, that is cleaved and processed to progressively smaller precursors [1, 24]. Ribosomopathies are often accompanied by defective or incomplete endo- and exo-nucleolytic cleavage of 18S, 5.8S and 28S rRNAs, encoded by the 47S precursor transcribed by RNA polymerase I [1, 6, 23]. A qRT-PCR-based assay to quantify these rRNA precursors (Fig 2A) showed evidence for their excessive accumulation, particularly in the case of those comprising the 18S-ITS1 junction (Fig 2B). An even more pronounced processing defect was seen in all tested HCCs (Fig 2C). Thus, both HBs and HCCs showed evidence for inefficient rRNA precursor processing.

### RP transcript deregulation and mutation in human cancers

**Fig 3:**
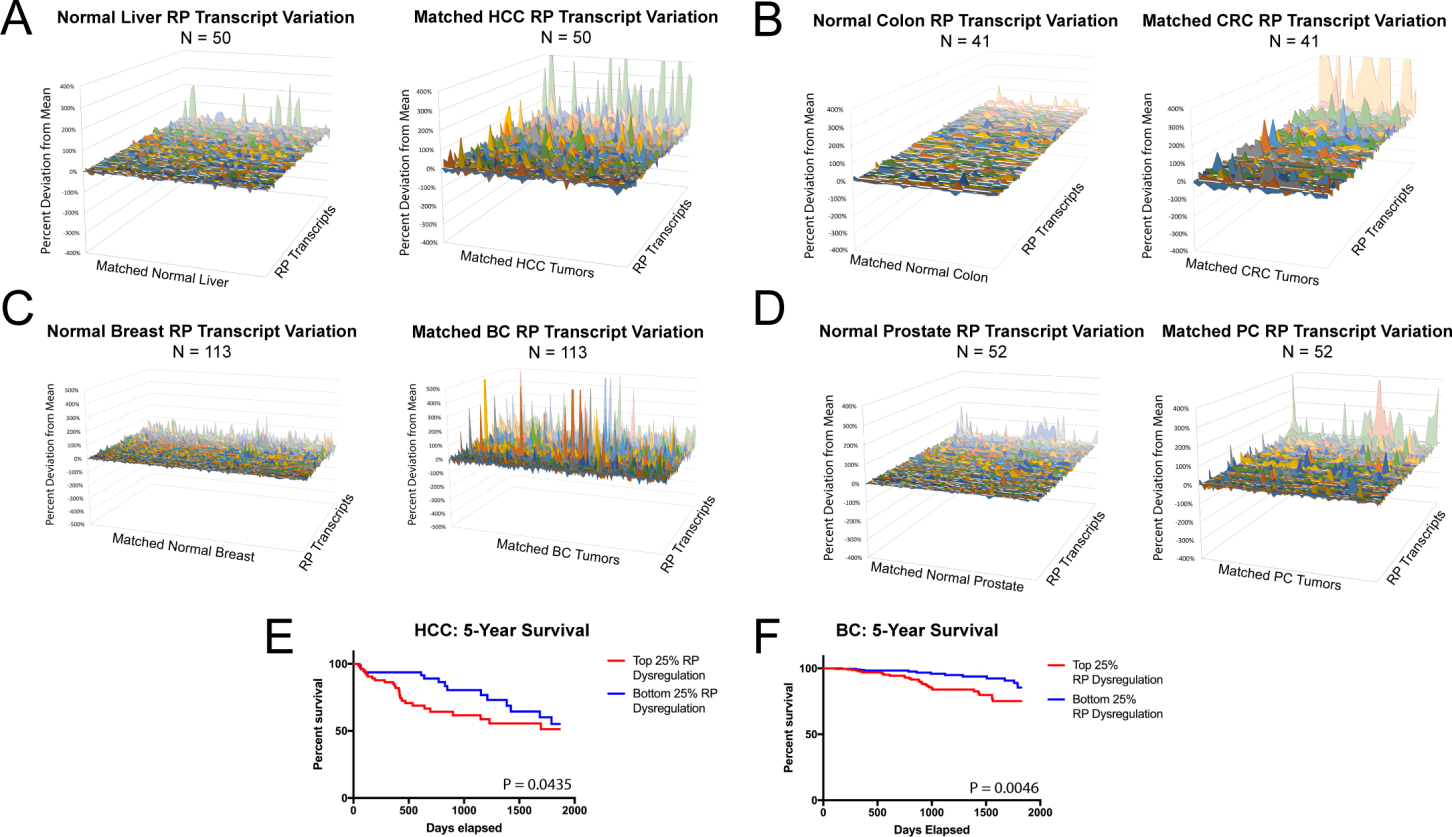
RP transcript deregulation in human cancers. 3D area maps of transcript levels for 77 RPs expressed in HCCs (A), CRCs (B), BCs (C) and PCs (D). To better evaluate differences in the other transcripts which did reach significance for their F-tests, these transcripts–*Rps26*, *Rpl9*, *Rps27*, *Rps28*, and *Rpl21*– were excluded from 3D area plots. For each cancer, tumors with matched samples of normal tissue in TCGA were selected for direct comparison (50 for HCC, 41 for CRC, 113 for BC and 52 for PC). Relative expression for each RP transcript was calculated as in Fig 1. See Figures D-G in S1 File for 3D area plots of the above matched tumor data together with additional data from unmatched tumor samples. (E, F) Patient survival in HCC and BC inversely correlates with the severity of RP transcript deregulation. Patients were sorted according to their RP transcript deregulation, and survival curves were plotted for the top and bottom 25% of patients with the greatest and least degree of RP transcript deregulation.

To explore the broader implications of our findings, we queried 2260 human cancers representing four major tumor types: HCC, colorectal cancer (CRC), breast cancer (BC) and prostate cancer (PC). Our rationale for choosing these particular cancers was that, like the above mouse tumor models, the first two are commonly associated with defects in Wnt-β-catenin and/or YAP signaling. All four types may also over-express Myc, which drives both RP transcript and rRNA biogenesis [15, 16, 25]. Deregulation of individual RPs or RP transcripts has been described sporadically in some human cancers and cancer cell lines, thereby suggesting more global underlying defects [26]. However, a systematic study has not to our knowledge been performed. As seen in Fig 3A–3D and Figures D-G in S1 File, RP transcript deregulation was common in each human cancer cohort. Deregulated transcripts were defined as any whose relative expression level fell significantly outside the mean distribution seen in normal tissues (P<0.05). By this definition, the average number of deregulated RP transcripts per tumor was 28.3 in HCCs, 26.2 in CRCs, 23.6 in BCs and 14.6 in PCs (P = 2.6x10^-17^, 2.8x10^-15^, 2.1x10^-12^ and 2.6x10^-5^, respectively). 25 RP transcripts were significantly deregulated in the same direction in three tumor types and 12 were deregulated in the same direction in all four types (Table B in S1 File). Moreover, for HCCs and BCs, the severity of deregulation correlated inversely with survival (Fig 3E and 3F).

We considered the possibility that RP transcript dysregulation in the above human cancers might be due to RP gene amplification or deletion. However, a query for copy number alterations (CNAs) in these cancer cohorts using cBioPortal (www.cbioportal.org) revealed that only 30.7–53.7% of tumors possessed a CNA in any RP gene. Moreover, those tumors which did harbor CNAs, contained an average of only 1.2–1.9 such variations per tumor. Thus, CNV does not explain the vast majority of RP transcript dysregulation reported here.

**Fig 4:**
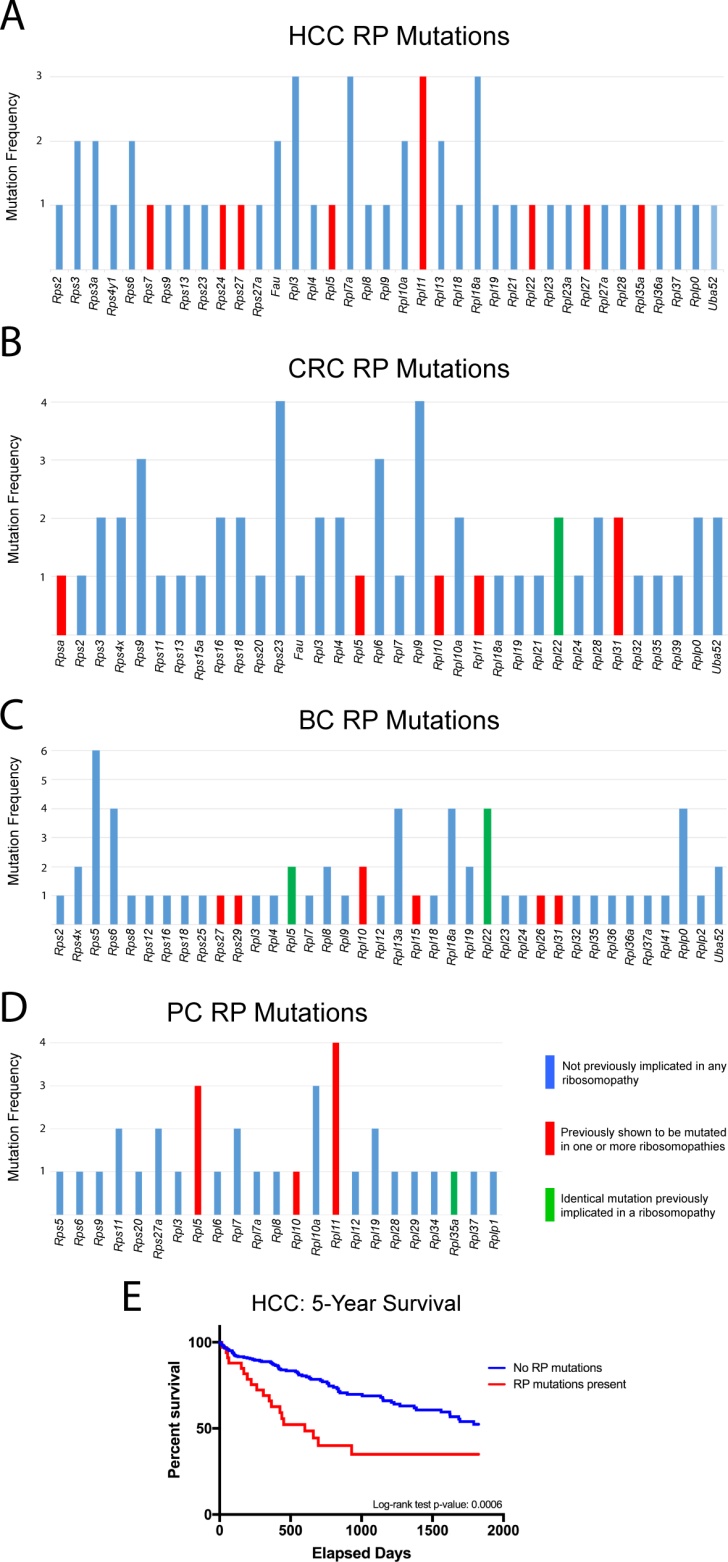
RP transcript mutations identified in human cancers. The results of RP mutational analysis were obtained from the sequence data shown in Fig 3. The total number of mutations involving any given RP are plotted along the ordinate. All mutations identified are listed individually in Tables F-G in S1 File. (A). HCCs. (B). Colo-rectal cancers (CRCs). (C). Breast cancers (BC). (D). Prostate cancers (PC). Identical mutations previously implicated in a ribosomopathy include: K89Nf2*3 and K15Rfs*5 in *Rpl22* in CRC; N57Efs*12 and A97G in *Rpl5*, and K15Rfs*5 in *Rpl22* in BC; and V33I in *Rpl35A* in PC. Previously described RP mutations were identified in refs. 1,2,5,6,9,43–45 and http://www.dbagenes.unito.it/home.php. (E). Survival of HCC patients with and without RP transcript coding mutations.

205 somatic RP mutations were detected in the above tumor types, as reported by cBioPortal (Fig 4A–4D). These involved 70 distinct RPs, at least 14 of which have been previously implicated in one or more ribosomopathy [1, 2, 10, 26, 27]. 116 of the mutations were recurrent or involved the same RP in at least two tumors. The most notable of these included *Rpl5* (7 mutations), *Rpl11* (8 mutations) and *Rpl22* (7 mutations). Nine mutations, identical to those described previously in ribosomopathies, were also identified. In total, 51 of 373 (14%) individuals with HCC harbored RP mutations and their survival was significantly inferior to that of individuals without mutations (Fig 4E).

We next asked whether tumors with RP point mutations might be associated with a higher frequency of mutations in known oncogenic drivers. To address this on a global scale, tumor mutation data for the above four TCGA human cancer cohorts were obtained using XenaBrowser (https://xenabrowser.net) and selecting “somatic non-silent mutation (gene-level) -> broad automated”. Tumors were grouped into those with and without RP coding region mutations. For each of the >40,000 genes listed in the tumor mutation databases, Chi-squared tests were performed to determine if the frequencies of non-RP gene mutations differed between the two tumor groups. Many thousands of genes were mutated at a significantly greater frequency in the RP mutant tumor groups in each of the four cohorts investigated, indicating that tumors with RP mutations were more likely to accumulate other mutations as well.

We then performed additional Chi-squared tests to determine whether any particular mutations occurred in RP mutant tumors at frequencies higher than expected if they were distributed randomly. In breast, liver, and prostate cancer, none of these subsequent Chi-squared tests were significant after Bonferroni false-discovery (FDR) correction. Thus, while RP mutant tumors were more likely to possess mutations in other genes as noted above, no specific gene(s) appeared to be favored.

In contrast, 42 genes were mutated in RP mutant CRCs at a higher frequency than expected (35–70% of RP mutant tumors versus 1%-32% in non-RP mutant tumors) (P < 0.05 after FDR-correction). However, none of the 42 genes were canonical oncogenes, tumor suppressors or otherwise known CRC drivers (Table H in S1 File). Taken together, these results indicate that, tumors with and without RP point mutations cannot be distinguished based on the identity or frequency of otherwise well-accepted driver mutations.

### Inactivation of the p19^ARF^/Mdm2/p53 pathway in liver cancer

Ribosomal stress, a hallmark of ribosomopathies [1, 10, 27], can activate the p19^*ARF*^/Mdm2/p53 tumor suppressor pathway and block proliferation at several points (Figure H in S1 File). For example, p19^*ARF*^ interferes with ribosome assembly by preventing the nucleolar export of 40S and 60S ribosomal subunits [28, 29]. p19^*ARF*^ also interacts with Mdm2 thereby inhibiting its E3 ubiquitin ligase activity and indirectly stabilizing p53 [3, 30, 31]. A subset of free RPs that cannot assemble into mature ribosomes in the face of RP haploinsufficiency also interact with Mdm2 and inhibit its interaction with p53 [1, 2, 32, 33]. Most, but not all, of these RPs interact with Mdm2 via the latter protein’s central acid domain [30]. Finally, RPS26, among the most reliably down-regulated RPs in HBs and HCCs (Fig 1C and 1D), de-stabilizes p53 and augments its transcriptional activation [34].

**Fig 5:**
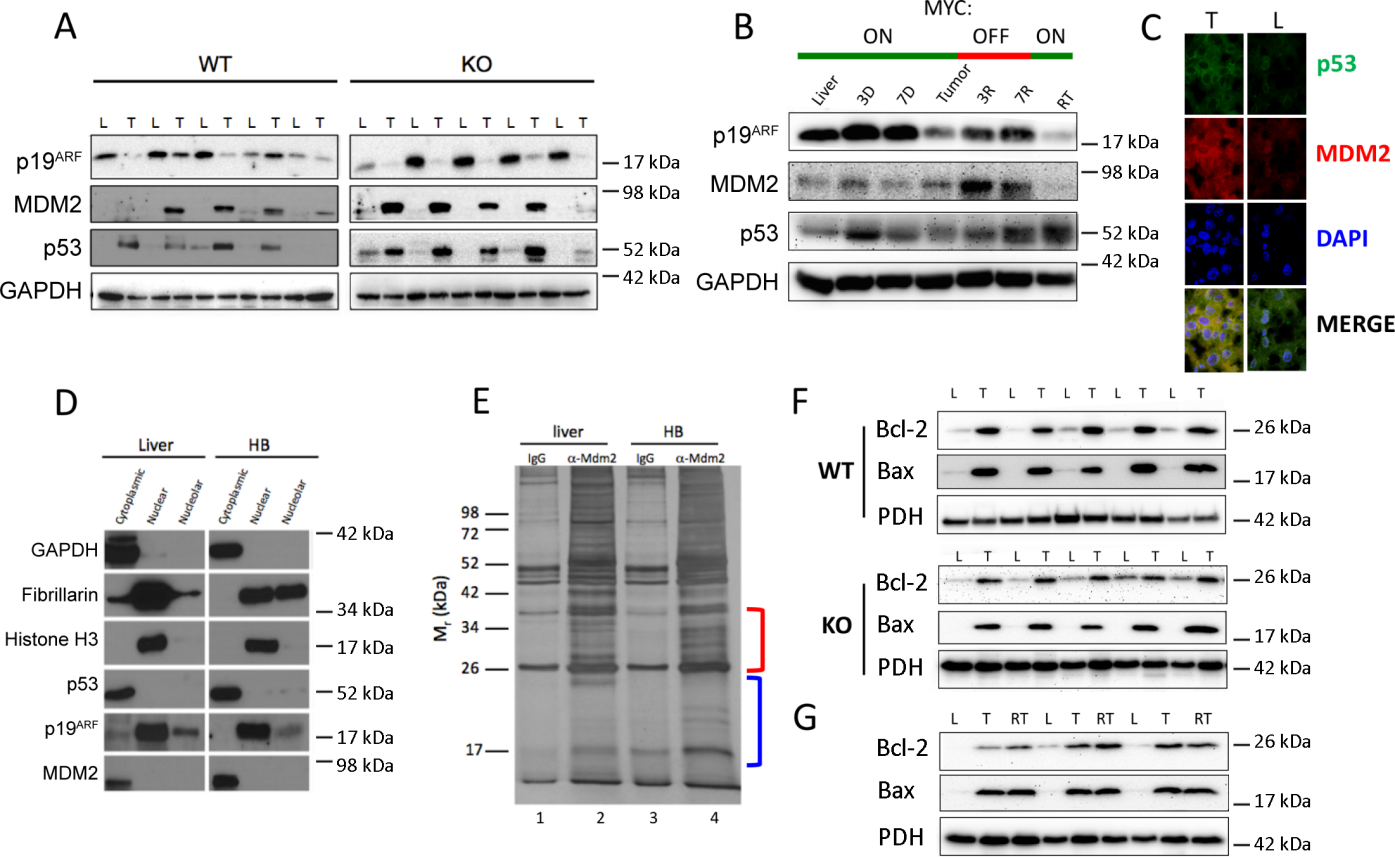
Reprograming of survival and apoptosis pathways in HBs and HCCs. (A) Expression of p19^ARF^, MDM2 and p53 in total liver (L) and HB (T) lysates from WT and KO mice. (B) Similar immuno-blots from HCCs. (C) Immuno-staining of frozen sections of liver (L) and WT HBs (T) for p53 and MDM2. Using ImageJ software (https://imagej.nih.gov/ij/), we determined that >80% of Mdm2 and p53 localized to the cytoplasm in both livers and tumors. (D) p53 and MDM2 co-localize to HB cytoplasm. A freshly collected WT HB tumor was fractionated into cytoplasmic, nuclear and nucleolar compartments. Each fraction was tested for the protein markers localizing to these compartments (GAPDH, histone H3 and fibrillarin, respectively) and in parallel for p53, p19^ARF^ and MDM2. Varying amounts of lysate and exposure times were required to compensate for differential protein expression. (E) Liver and HB cytoplasmic fractions were immuno-precipitated with control IgG or anti-MDM2 IgG. Precipitates were resolved by SDS-PAGE and silver stained. Bracketed regions were excised from lanes 2 and 4 and subjected to trypsin digestion and mass spectrometry. (F) Bcl-2 and Bax expression in mitochondria from WT or KO HBs [16]. The same blot was probed with an antibody for the mitochondrial protein pyruvate dehydrogenase E1α subunit (PDH) as a control for protein loading. The mean up-regulation of Bcl-2 relative to that in livers was 3.8-fold in WT HBs and 2.3-fold in KO HBs. The mean up-regulation of Bax was 11.7-fold in WT HBs and 10.5-fold in KO HBs. (G) Bcl-2 and Bax expression in isolated mitochondria from livers (L), tumors (T) and recurrent HCC tumors [12]. The mean up-regulation of Bcl-2 was 5.5-fold in initial tumors and 6.3-fold in recurrent tumors. Similarly, the mean up-regulation of Bax was 15-fold in initial tumors and 15.6-fold in recurrent tumors.

Defects affecting the expression and/or subcellular localization of p19^*ARF*^/Mdm2/p53 pathway members could account for how murine HBs and HCCs escape the growth inhibition mediated by RP deregulation [1, 2, 4, 10]. Indeed, p19^*ARF*^ protein was markedly down-regulated in most WT and KO HBs whereas Mdm2 and p53 were up-regulated (Fig 5A). In HCCs, p19^*ARF*^ was also reduced in both initial and recurrent tumors and in some cases normalized during early regression. Mdm2, while detectable at all times, was elevated in regressing tumors whereas p53 was generally expressed at low levels in both tumors and livers (Fig 5B and Figure I in S1 File). Immuno-staining of frozen liver and HB sections and quantification using Image J software (https://imagej.nih.gov/ij/) showed >80% Mdm2 and p53 to be cytoplasmically localized in both (Fig 5C). These results were confirmed by subcellular fractionation studies showing that Mdm2 and p53 in HBs co-localized to the cytoplasm as did the small amounts of p19^*ARF*^ that remained detectable (Fig 5D). *Trp53* mutations, which can also affect p53 protein stability [35, 36], were not identified upon sequencing of transcripts from 14 WT and KO HBs and 10 primary and recurrent HCCs.

In order to demonstrate that the down-regulation of p19^*ARF*^ was necessary for HB tumor induction, we repeated HDTV injections with SB vectors encoding mutant β-catenin and YAP but included an additional SB vector encoding p19^*ARF*^ or, in control animals, the empty SB vector only. As expected, all 10 mice in the control group succumbed to large HBs within 12–15 wks, whereas none of the 10 mice co-inoculated with p19^*ARF*^ developed tumors after as long as 30 wks and had normal sized livers when the study was terminated. Thus, restoring normal levels of p19^*ARF*^ expression strongly inhibits tumorigenesis indicating that disruption of the p19^*ARF*^/Mdm2/p53 pathway is an essential “second hit”.

Attempts to co-immuno-precipitate (co-IP) Mdm2-p53 complexes from HB lysates using several anti-Mdm2 and anti-p53 antibodies were unsuccessful, thereby suggesting that the proteins were not interacting despite their common cytoplasmic residency (Fig 5C). This further suggested that, by analogy to the ribosomopathies, free RPs might be binding Mdm2 and impairing its interaction with p53 [1, 2, 10, 27, 32]. IPs of liver and HB cytoplasmic compartments with an anti-Mdm2 antibody followed by SDS-PAGE and silver staining showed distinct bands otherwise absent from control IPs performed with non-immune IgG as well as differences between anti-Mdm2 IPs from liver and HBs (Fig 5E). Two regions of these gels, corresponding to M_r_s ~24–35 kDa and ~14–24 kDa (Fig 5E, red and blue brackets, respectively) were excised and subjected to tryptic digest and mass spectrometry. In the first case, we identified 12 RPs from the HB lane and 7 RPs from the control liver lane. All of the latter RPs were also identified in the former sample (Table C and Figures K-L in S1 File). RPs detected in the ~14–24 kDa samples included 17 in the HB IP and 11 in the liver IP. All but one of the RPs in the latter group was also detected in the former group (Table D and Figures M-N in S1 File). Overall, 5 of 30 RPs identified by this analysis (RPSA, RPL11, RPS10, RPS17 and RPS24) have been previously implicated in ribosomopathies [1–4, 7, 10, 26, 27, 30]. Thus, in primary HBs, Mdm2 binds a larger subset of RPs than it does in normal livers. This supports the idea that free RPs bind to Mdm2 and block p53 binding [1, 4, 26, 30]. Co-IPs from similarly fractionated HCC cytoplasmic extracts (Figure J in S1 File) identified eight Mdm2-interacting RPs in common with HB as well as an additional nine unique to HCCs (Figures O-P and Table E in S1 File).

Nuclear exclusion of wild-type p53 as shown in Fig 5C and 5D could explain how tumors escape growth inhibition and apoptosis, which require substantial transcriptional re-programming [35, 36]. However, p53 can also promote apoptosis via transcription-independent mitochondrial pathways [36, 37] that can be blocked by Bcl-2 [38]. Indeed, both HBs and initial and recurrent HCCs expressed higher levels of mitochondrial Bcl-2 suggesting that this anti-apoptotic protein mitigates any extra-nuclear p53 functions (Fig 5F and 5G). On the other hand, these same tumors also showed similarly elevated levels of the pro-apoptotic protein Bax. Taken together, these results suggest that, in the case of both HBs and HCCs, any apoptotic predisposition is mitigated at the nuclear level by p53’s inability to transcriptionally activate cell death-related genes and at the mitochondrial level by the up-regulation of Bcl-2, which interferes with Bax-mediated loss of mitochondrial integrity.

## Discussion

The number and heterogeneity of RP abnormalities described here currently preclude a precise determination as to how any individual RP contributes to cancer pathogenesis. Indeed, whether this is even attributable solely to altered protein translation [26, 27] is uncertain in light of accumulating evidence that RPs engage in numerous extra-ribosomal functions such as transcription, cell cycle regulation, DNA damage repair and survival and that some deregulated RPs are themselves oncogenic or can suppress the actions of chemotherapeutic agents such as actinomycin D and 5-fluorouracil [4, 39–41]. The loss of the normal stoichiometric relationships between RPs and certain translation initiation factors such as eIF3 and eIF4e, which may be transforming when deregulated [42], might also conceivably abet transformation as might the assembly of abnormal ribosomes with different compositions and/or modifications [43]. The deregulation of oncoproteins such as Myc and Bcl-2 and tumor suppressors such as p53 might also contribute to RP abnormalities during tumor evolution with the relative importance of these factors being further dictated by both tissue and tumor heterogeneity [27, 44]. What is clear however, is that, regardless of the underlying cause(s) of RP transcript and RP de-regulation, the consequences involve many of the same mechanisms and pathways that have been previously described in the ribosomopathies, where they are thought to underlie disease pathogenesis and cancer susceptibility [1–4]. These include a massive and much more pronounced deregulation of RP transcripts and proteins than occurs in classical ribosomopathies and a ribosomopathy-like maturation defect in rRNA processing in both of the animal models we have studied. It also seems likely that RP deregulation and the direct binding of numerous free RPs to Mdm2 precludes the latter protein’s interaction with and subsequent ubiquitin-mediated degradation of p53.

Despite these similarities, a major difference between the cancers discussed here and classical ribosomopathies is that the latter disorders are believed to be associated with p53-mediated bone marrow suppression [1–4]. The fact that p53 remains detectable and non-mutated in HBs and HCCs without exerting any apparent growth inhibitory effects is likely attributable to a combination of its cytoplasmic localization and the additional protective effect of Bcl-2 up-regulation, despite an equally high concurrent up-regulation of Bax (Fig 5F). The combination of high-level Myc (or N-Myc) and Bcl2 expression has been previously shown to prevent the nuclear translocation of otherwise wild-type p53 in both hematopoietic and non-hematopoietic cell types [45–47]. In addition, the otherwise pro-apoptotic effect of increased Bax expression in both HBs and HCCs (Fig 5F) may be mitigated by the fact that these tumors, as well as their corresponding normal livers, express undetectable or barely detectable levels of caspase 2 (not shown), which is necessary for the execution of Bax-mediated mitochondrial apoptosis [37, 48].

In the era prior to the discovery of oncogenes and tumor suppressors, Dameshek first remarked upon the seemingly paradoxical relationship between hypoplastic hematopoietic disorders and susceptibility to leukemia [49]. Appropriating a term from bacterial genetics, he proposed that surviving hematopoietic cells might acquire clonal “suppressor” mutations, which allow them to overcome their intrinsic proliferative block and become transformed. The results with two murine cancer models described here have identified some of these potential suppressors and provide mechanistic insights into how the tendency toward cell cycle arrest and apoptosis arising from ribosomal stress can be circumvented. Not surprisingly, this involves what are now regarded to be the same canonical oncogenic and tumor suppressor pathways believed to operate in more “classical” ribosomopathies [1, 27]. Such mechanisms might explain why some human tumors, including HB and HCC, often express high levels of wild-type p53 and occasionally co-express Bcl-2 and how p53 may at times actually facilitate tumor survival [36, 50–53]. Given the heterogeneity of RP and transcript deregulation in both classical ribosomopathies and sporadic human cancers (Fig 3), it seems likely that other compensatory mutations exist, including those involving p53 and p19^*ARF*^ inactivation and Mdm2 over-expression.

In summary, experimentally generated murine HBs and HCCs are associated with frequent and heretofore unrecognized RP transcript and protein deregulation. Similar findings regarding RP transcripts were made in four common human cancer types with the severity of transcript deregulation and/or the presence of mutations correlating inversely with survival in two of the three cohorts in which such analysis was possible. Although rare, some human cancer-associated RP point mutations were identical to those previously described in ribosomopathies, thus implying a causal contribution. It remains to be determined how many of the remaining mutations, not previously identified in ribosomopathies, have functional consequences given that they do not appear to represent polymorphisms [54].

As is also common in ribosomopathies, defective rRNA processing was observed in our murine tumor models. Compensatory mechanisms to evade growth inhibitory signals arising from the ensuing ribosomal stress center around p19^*ARF*^ silencing, the cytoplasmic sequestration of p53, and the induction of Bcl-2. Ribosomopathies, originally identified as rare, mostly inborn disorders of hematopoiesis and development [1, 2, 10, 27], now appear to include many human cancers with implications that remain to be determined.

## Supporting information

S1 File Supporting information for Materials and methods, Figures A-P and legends and Tables A-H and legends.
(PDF)

## References

[1] Ruggero D, Shimamura A. Marrow failure: a window into ribosome biology. Blood. 2014;124(18):2784–92. doi: 10.1182/blood-2014-04-526301 ; PubMed Central PMCID: PMC4215310. 25237201 10.1182/blood-2014-04-526301 PMC4215310

[2] Yelick PC, Trainor PA. Ribosomopathies: Global process, tissue specific defects. Rare Diseases. 2015;3(1):e1025185 doi: 10.1080/21675511.2015.1025185 26442198 10.1080/21675511.2015.1025185 PMC4590025

[3] Boultwood J, Pellagatti A, Wainscoat JS. Haploinsufficiency of ribosomal proteins and p53 activation in anemia: Diamond-Blackfan anemia and the 5q- syndrome. Adv Biol Regul. 2012;52(1):196–203. doi: 10.1016/j.advenzreg.2011.09.008 . 21930148 10.1016/j.advenzreg.2011.09.008

[4] Shenoy N, Kessel R, Bhagat TD, Bhattacharyya S, Yu Y, McMahon C, et al Alterations in the ribosomal machinery in cancer and hematologic disorders. J Hematol Oncol. 2012;5:32 doi: 10.1186/1756-8722-5-32 ; PubMed Central PMCID: PMC3438023. 22709827 10.1186/1756-8722-5-32 PMC3438023

[5] De Keersmaecker K, Atak ZK, Li N, Vicente C, Patchett S, Girardi T, et al Exome sequencing identifies mutation in CNOT3 and ribosomal genes RPL5 and RPL10 in T-cell acute lymphoblastic leukemia. Nature Genetics. 2012;45(2):186–90. doi: 10.1038/ng.2508 23263491 10.1038/ng.2508 PMC5547913

[6] Gazda HT, Preti M, Sheen MR, O'Donohue MF, Vlachos A, Davies SM, et al Frameshift mutation in p53 regulator RPL26 is associated with multiple physical abnormalities and a specific pre-ribosomal RNA processing defect in diamond-blackfan anemia. Hum Mutat. 2012;33(7):1037–44. doi: 10.1002/humu.22081 ; PubMed Central PMCID: PMC3370062. 22431104 10.1002/humu.22081 PMC3370062

[7] Burwick N, Shimamura A, Liu JM. Non-Diamond Blackfan anemia disorders of ribosome function: Shwachman Diamond syndrome and 5q- syndrome. Semin Hematol. 2011;48(2):136–43. doi: 10.1053/j.seminhematol.2011.01.002 ; PubMed Central PMCID: PMC3072806. 21435510 10.1053/j.seminhematol.2011.01.002 PMC3072806

[8] Russo A, Russo G. Ribosomal Proteins Control or Bypass p53 during Nucleolar Stress. Int J Mol Sci. 2017;18(1). doi: 10.3390/ijms18010140 ; PubMed Central PMCID: PMC5297773. 28085118 10.3390/ijms18010140 PMC5297773

[9] Fumagalli S, Ivanenkov VV, Teng T, Thomas G. Suprainduction of p53 by disruption of 40S and 60S ribosome biogenesis leads to the activation of a novel G2/M checkpoint. Genes Dev. 2012;26(10):1028–40. doi: 10.1101/gad.189951.112 ; PubMed Central PMCID: PMC3360559. 22588717 10.1101/gad.189951.112 PMC3360559

[10] De Keersmaecker K, Sulima SO, Dinman JD. Ribosomopathies and the paradox of cellular hypo- to hyperproliferation. Blood. 2015;125(9):1377–82. doi: 10.1182/blood-2014-10-569616 ; PubMed Central PMCID: PMC4342353. 25575543 10.1182/blood-2014-10-569616 PMC4342353

[11] Jung KY, Wang H, Teriete P, Yap JL, Chen L, Lanning ME, et al Perturbation of the c-Myc-Max protein-protein interaction via synthetic alpha-helix mimetics. J Med Chem. 2015;58(7):3002–24. doi: 10.1021/jm501440q ; PubMed Central PMCID: PMC4955407. 25734936 10.1021/jm501440q PMC4955407

[12] Hannan KM, Brandenburger Y, Jenkins A, Sharkey K, Cavanaugh A, Rothblum L, et al mTOR-dependent regulation of ribosomal gene transcription requires S6K1 and is mediated by phosphorylation of the carboxy-terminal activation domain of the nucleolar transcription factor UBF. Mol Cell Biol. 2003;23(23):8862–77. doi: 10.1128/MCB.23.23.8862-8877.2003 ; PubMed Central PMCID: PMC262650. 14612424 10.1128/MCB.23.23.8862-8877.2003 PMC262650

[13] Shachaf CM, Kopelman AM, Arvanitis C, Karlsson Å, Beer S, Mandl S, et al MYC inactivation uncovers pluripotent differentiation and tumour dormancy in hepatocellular cancer. Nature. 2004;431(7012):1112–7. doi: 10.1038/nature03043 15475948 10.1038/nature03043

[14] Tao J, Calvisi DF, Ranganathan S, Cigliano A, Zhou L, Singh S, et al Activation of β-Catenin and Yap1 in Human Hepatoblastoma and Induction of Hepatocarcinogenesis in Mice. Gastroenterology. 2014;147(3):690–701. doi: 10.1053/j.gastro.2014.05.004 24837480 10.1053/j.gastro.2014.05.004 PMC4143445

[15] Kim S, Li Q, Dang CV, Lee LA. Induction of ribosomal genes and hepatocyte hypertrophy by adenovirus-mediated expression of c-Myc in vivo. Proceedings of the National Academy of Sciences. 2000;97(21):11198–202. doi: 10.1073/pnas.200372597 11005843 10.1073/pnas.200372597 PMC17177

[16] Wang H, Lu J, Edmunds LR, Kulkarni S, Dolezal J, Tao J, et al Coordinated Activities of Multiple Myc-Dependent and Myc-Independent Biosynthetic Pathways in Hepatoblastoma. Journal of Biological Chemistry. 2016:jbc.M116.754218. doi: 10.1074/jbc.m116.754218 27738108 10.1074/jbc.M116.754218 PMC5159488

[17] Edmunds LR, Otero PA, Sharma L, D’Souza S, Dolezal JM, David S, et al Abnormal lipid processing but normal long-term repopulation potential of *myc*-/- hepatocytes. Oncotarget. 2015 doi: 10.18632/oncotarget.8856 27105497 10.18632/oncotarget.8856 PMC5058687

[18] Chen X, Calvisi DF. Hydrodynamic Transfection for Generation of Novel Mouse Models for Liver Cancer Research. The American Journal of Pathology. 2014;184(4):912–23. doi: 10.1016/j.ajpath.2013.12.002 24480331 10.1016/j.ajpath.2013.12.002 PMC3969989

[19] Dolezal JM, Wang H, Kulkarni S, Jackson L, Lu J, Ranganathan S, et al Sequential Adaptive Changes in a c-Myc-Driven Model of Hepatocellular Carcinoma. The Journal of biological chemistry. 2017 Epub 2017/04/23. doi: 10.1074/jbc.M117.782052 . 28432125 10.1074/jbc.M117.782052 PMC5473214

[20] Shevchenko A, Tomas H, Havli J, Olsen JV, Mann M. In-gel digestion for mass spectrometric characterization of proteins and proteomes. Nature Protocols. 2007;1(6):2856–60. doi: 10.1038/nprot.2006.468 17406544 10.1038/nprot.2006.468

[21] Perry RP. Balanced production of ribosomal proteins. Gene. 2007;401(1–2):1–3. doi: 10.1016/j.gene.2007.07.007 ; PubMed Central PMCID: PMC5370545. 17689889 10.1016/j.gene.2007.07.007 PMC5370545

[22] Strunk BS, Novak MN, Young CL, Karbstein K. A translation-like cycle is a quality control checkpoint for maturing 40S ribosome subunits. Cell. 2012;150(1):111–21. doi: 10.1016/j.cell.2012.04.044 ; PubMed Central PMCID: PMC3615461. 22770215 10.1016/j.cell.2012.04.044 PMC3615461

[23] Tafforeau L, Zorbas C, Langhendries J-L, Mullineux S-T, Stamatopoulou V, Mullier R, et al The Complexity of Human Ribosome Biogenesis Revealed by Systematic Nucleolar Screening of Pre-rRNA Processing Factors. Molecular Cell. 2013;51(4):539–51. doi: 10.1016/j.molcel.2013.08.011 23973377 10.1016/j.molcel.2013.08.011

[24] Tafforeau L, Zorbas C, Langhendries JL, Mullineux ST, Stamatopoulou V, Mullier R, et al The complexity of human ribosome biogenesis revealed by systematic nucleolar screening of Pre-rRNA processing factors. Mol Cell. 2013;51(4):539–51. doi: 10.1016/j.molcel.2013.08.011 . 23973377 10.1016/j.molcel.2013.08.011

[25] Gomez-Roman N, Felton-Edkins ZA, Kenneth NS, Goodfellow SJ, Athineos D, Zhang J, et al Activation by c-Myc of transcription by RNA polymerases I, II and III. Biochem Soc Symp. 2006;73:141–54. doi: 10.1042/bss0730141 10.1042/bss0730141 16626295

[26] Goudarzi K, Lindström M. Role of ribosomal protein mutations in tumor development (Review). Int J Oncol. 2016 doi: 10.3892/ijo.2016.3387 26892688 10.3892/ijo.2016.3387 PMC4777597

[27] Armistead J, Triggs-Raine B. Diverse diseases from a ubiquitous process: The ribosomopathy paradox. FEBS Letters. 2014;588(9):1491–500. doi: 10.1016/j.febslet.2014.03.024 24657617 10.1016/j.febslet.2014.03.024

[28] Apicelli AJ, Maggi LB, Hirbe AC, Miceli AP, Olanich ME, Schulte-Winkeler CL, et al A Non-Tumor Suppressor Role for Basal p19ARF in Maintaining Nucleolar Structure and Function. Molecular and Cellular Biology. 2007;28(3):1068–80. doi: 10.1128/MCB.00484-07 18070929 10.1128/MCB.00484-07 PMC2223401

[29] Bertwistle D, Sugimoto M, Sherr CJ. Physical and Functional Interactions of the Arf Tumor Suppressor Protein with Nucleophosmin/B23. Molecular and Cellular Biology. 2004;24(3):985–96. doi: 10.1128/MCB.24.3.985-996.2004 14729947 10.1128/MCB.24.3.985-996.2004 PMC321449

[30] Kim TH, Leslie P, Zhang Y. Ribosomal proteins as unrevealed caretakers for cellular stress and genomic instability. Oncotarget. 2014;5(4):860–71. doi: 10.18632/oncotarget.1784 ; PubMed Central PMCID: PMC4011588. 24658219 10.18632/oncotarget.1784 PMC4011588

[31] Weber JD, Kuo ML, Bothner B, DiGiammarino EL, Kriwacki RW, Roussel MF, et al Cooperative signals governing ARF-mdm2 interaction and nucleolar localization of the complex. Mol Cell Biol. 2000;20(7):2517–28. ; PubMed Central PMCID: PMC85460. 10713175 10.1128/mcb.20.7.2517-2528.2000 PMC85460

[32] Bursac S, Brdovcak MC, Pfannkuchen M, Orsolic I, Golomb L, Zhu Y, et al Mutual protection of ribosomal proteins L5 and L11 from degradation is essential for p53 activation upon ribosomal biogenesis stress. Proc Natl Acad Sci U S A. 2012;109(50):20467–72. doi: 10.1073/pnas.1218535109 ; PubMed Central PMCID: PMC3528581. 23169665 10.1073/pnas.1218535109 PMC3528581

[33] Macias E, Jin A, Deisenroth C, Bhat K, Mao H, Lindström MS, et al An ARF-Independent c-MYC-Activated Tumor Suppression Pathway Mediated by Ribosomal Protein-Mdm2 Interaction. Cancer Cell. 2010;18(3):231–43. doi: 10.1016/j.ccr.2010.08.007 20832751 10.1016/j.ccr.2010.08.007 PMC4400806

[34] Cui D, Li L, Lou H, Sun H, Ngai SM, Shao G, et al The ribosomal protein S26 regulates p53 activity in response to DNA damage. Oncogene. 2013;33(17):2225–35. doi: 10.1038/onc.2013.170 23728348 10.1038/onc.2013.170

[35] Bálint É, Vousden KH. Activation and activities of the p53 tumour suppressor protein. British Journal of Cancer. 2001;85(12):1813–23. doi: 10.1054/bjoc.2001.2128 11747320 10.1054/bjoc.2001.2128 PMC2364002

[36] Kruiswijk F, Labuschagne CF, Vousden KH. p53 in survival, death and metabolic health: a lifeguard with a licence to kill. Nature Reviews Molecular Cell Biology. 2015;16(7):393–405. doi: 10.1038/nrm4007 26122615 10.1038/nrm4007

[37] Jiang M, Milner J. Bcl-2 constitutively suppresses p53-dependent apoptosis in colorectal cancer cells. Genes Dev. 2003;17(7):832–7. doi: 10.1101/gad.252603 ; PubMed Central PMCID: PMC196025. 12670866 10.1101/gad.252603 PMC196025

[38] Chiou SK, Rao L, White E. Bcl-2 blocks p53-dependent apoptosis. Molecular and Cellular Biology. 1994;14(4):2556–63. doi: 10.1128/mcb.14.4.2556 8139558 10.1128/mcb.14.4.2556-2563.1994 PMC358623

[39] Amsterdam A, Sadler KC, Lai K, Farrington S, Bronson RT, Lees JA, et al Many Ribosomal Protein Genes Are Cancer Genes in Zebrafish. PLoS Biology. 2004;2(5):e139 doi: 10.1371/journal.pbio.0020139 15138505 10.1371/journal.pbio.0020139 PMC406397

[40] Russo A, Pagliara V, Albano F, Esposito D, Sagar V, Loreni F, et al Regulatory role of rpL3 in cell response to nucleolar stress induced by Act D in tumor cells lacking functional p53. Cell Cycle. 2016;15(1):41–51. doi: 10.1080/15384101.2015.1120926 ; PubMed Central PMCID: PMC4825706. 26636733 10.1080/15384101.2015.1120926 PMC4825706

[41] Russo A, Saide A, Cagliani R, Cantile M, Botti G, Russo G. rpL3 promotes the apoptosis of p53 mutated lung cancer cells by down-regulating CBS and NFkappaB upon 5-FU treatment. Sci Rep. 2016;6:38369 doi: 10.1038/srep38369 ; PubMed Central PMCID: PMC5141482. 27924828 10.1038/srep38369 PMC5141482

[42] Siddiqui N, Sonenberg N. Signalling to eIF4E in cancer. Biochemical Society Transactions. 2015;43(5):763–72. doi: 10.1042/BST20150126 26517881 10.1042/BST20150126 PMC4613458

[43] Truitt Morgan L, Conn Crystal S, Shi Z, Pang X, Tokuyasu T, Coady Alison M, et al Differential Requirements for eIF4E Dose in Normal Development and Cancer. Cell. 2015;162(1):59–71. doi: 10.1016/j.cell.2015.05.049 26095252 10.1016/j.cell.2015.05.049 PMC4491046

[44] Dai M-S, Lu H. Crosstalk between c-Myc and ribosome in ribosomal biogenesis and cancer. Journal of Cellular Biochemistry. 2008;105(3):670–7. doi: 10.1002/jcb.21895 18773413 10.1002/jcb.21895 PMC2569974

[45] Goldman SC, Chen CY, Lansing TJ, Gilmer TM, Kastan MB. The p53 signal transduction pathway is intact in human neuroblastoma despite cytoplasmic localization. Am J Pathol. 1996;148(5):1381–5. ; PubMed Central PMCID: PMC1861565. 8623910 PMC1861565

[46] Ryan JJ, Prochownik E, Gottlieb CA, Apel IJ, Merino R, Nunez G, et al c-myc and bcl-2 modulate p53 function by altering p53 subcellular trafficking during the cell cycle. Proc Natl Acad Sci U S A. 1994;91(13):5878–82. ; PubMed Central PMCID: PMC44100. 8016082 10.1073/pnas.91.13.5878 PMC44100

[47] Moll UM, LaQuaglia M, Benard J, Riou G. Wild-type p53 protein undergoes cytoplasmic sequestration in undifferentiated neuroblastomas but not in differentiated tumors. Proc Natl Acad Sci U S A. 1995;92(10):4407–11. ; PubMed Central PMCID: PMC41953. 7753819 10.1073/pnas.92.10.4407 PMC41953

[48] Matt S, Hofmann TG. The DNA damage-induced cell death response: a roadmap to kill cancer cells. Cell Mol Life Sci. 2016;73(15):2829–50. doi: 10.1007/s00018-016-2130-4 . 26791483 10.1007/s00018-016-2130-4 PMC11108532

[49] Dameshek W. Riddle: what do aplastic anemia, paroxysmal nocturnal hemoglobinuria (PNH) and "hypoplastic" leukemia have in common? Blood. 1967;30(2):251–4. . 6031145

[50] Bourdon J-C, D'Errico A, Paterlini P, Grigioni W, May E, Debuire B. p53 protein accumulation in European hepatocellular carcinoma is not always dependent on p53 gene mutation. Gastroenterology. 1995;108(4):1176–82. doi: 10.1016/0016-5085(95)90217-1 7698586 10.1016/0016-5085(95)90217-1

[51] Houben R, Hesbacher S, Schmid CP, Kauczok CS, Flohr U, Haferkamp S, et al High-Level Expression of Wild-Type p53 in Melanoma Cells is Frequently Associated with Inactivity in p53 Reporter Gene Assays. PLoS ONE. 2011;6(7):e22096 doi: 10.1371/journal.pone.0022096 21760960 10.1371/journal.pone.0022096 PMC3132323

[52] Piris MA, Pezzella F, Martinez-Montero JC, Orradre JL, Villuendas R, Sanchez-Beato M, et al p53 and bcl-2 expression in high-grade B-cell lymphomas: correlation with survival time. British Journal of Cancer. 1994;69(2):337–41. doi: 10.1038/bjc.1994.61 8297731 10.1038/bjc.1994.61 PMC1968699

[53] Rubio M-P, Louis DN. Accumulation of wild type p53 protein in human astrocytomas. Journal of Neuropathology and Experimental Neurology. 1993;52(3):323 doi: 10.1097/00005072-199305000-00252

[54] Lek M, Karczewski KJ, Minikel EV, Samocha KE, Banks E, Fennell T, et al Analysis of protein-coding genetic variation in 60,706 humans. Nature. 2016;536(7616):285–91. doi: 10.1038/nature19057 27535533 10.1038/nature19057 PMC5018207

