# Supplementary material for: Ribosomopathy-like properties of murine and human cancers
Source: PLoS One. 2017 Aug 18;12(8):e0182705. doi: 10.1371/journal.pone.0182705 (PMC5562309; doi:10.1371/journal.pone.0182705)
Supplement: S1 File — Supporting information for Materials and methods, Figures A-P and legends and Tables A-H and legends. (PDF) [file pone.0182705.s001.pdf]

S1 File: Supporting Information

Ribosomopathy-like Properties of Murine and Human Cancers

Sucheta Kulkarni[1,Ω], James M. Dolezal[1,Ω], Huabo Wang[1], Laura Jackson[2], Jie Lu[1], Brian P. Frodey[1], Atinuke Dosunmu-Ogunbi[1], Youjun Li[3], Marc Fromherz[1], Audry Kang[1,Δ], Lucas Santana-Santos[4], Panayiotis V. Benos[4,5] and Edward V. Prochownik[1,6,7,*]

Materials and methods

Selecting murine ribosomal proteins

The Mouse Genomics Informatics (MGI) website (http://www.informatics.jax.org) was queried for genes of type "protein coding gene" containing the name "ribosomal protein" without the strings "mitochondrial," "kinase," or "modification." Ninety genes were identified, but two (*Rpl32l and Rpl6l*) were not found in the RNA-seq cufflinks output for the hepatoblastoma analysis. Three additional genes (*Rpl10l, Rpl39l, Rpl3l*) were excluded from the HB analysis due to insufficient counts, resulting in a list of 85 mouse cytoplasmic ribosomal proteins. A total of 82 cytoplasmic RP genes were included in the HCC analysis, as eight of the 90 total cytoplasmic RP genes possessed insufficient counts in the RNA-seq output.

Accessing TCGA RNA-seq and clinical data

TCGA data was accessed through the University of California Santa Cruz Cancer Browser (https://genome-cancer.ucsuu.edu/proj/site/hgHeatmap/), downloading data listed under "gene expression (IlluminaHiSeq)" for each tumor type. Matched normal tissues for each cancer were identified by the following: 1) possessing at least two samples in the downloaded database,

one identified as “primary tumor” and one identified as “solid tissue normal” in the column “sample type”, and 2) presence of RNA-seq data for both samples. A list of 80 human ribosomal proteins was assembled from the University of Miyazaki’s Ribosomal Protein Gene Database (http://ribosome.med.miyazaki-u.ac.jp). When queried, three of these gene transcripts (*RPL40, RPS30, and RPS4Y*) were not found in the genomic data, so the final list used for the following analysis consisted of 77 RP genes. TCGA expression data, which is stored log-transformed, was base-two exponentiated for all samples.

TCGA: survival curves

To determine if RP transcript deregulation correlated with survival, this information was combined with clinical data from TCGA regarding days to death or last follow-up. Tumor samples meeting the following criteria were excluded from the survival analysis: 1) samples without corresponding clinical information, 2) samples with no recorded “days to death” or “days to last follow up”, or 3) days to death or last follow-up less than or equal to zero. There were 357 tumor samples in the HCC cohort with corresponding clinical information, 25 of which were excluded from the survival analysis on these criteria. The CRC cohort contained 278 tumor samples with clinical information and 4 were excluded. The BC cohort contained 1082 tumors with clinical information, 18 of which were excluded. Survival analysis was not performed for the PC cohort, as the requisite clinical information was available for only 6 patients. Tumor samples were then sorted according to the severity of RP transcript deregulation and placed into the upper and lower quartiles. There were 83 tumors per quartile in the HCC cohort, 69 tumors per quartile in the CRC cohort, and 266 tumors per quartile in the BC cohort. Five-year survival curves were generated comparing the top and bottom quartiles in each cohort, with significance

determined by a Log-rank test P-value < 0.05. Survival differences were significant in HCC (P = 0.0435) and BC (P = 0.0046).

TCGA: mutation analysis

TCGA mutation information was accessed using cBioPortal (http://www.cbioportal.org/), from the "TCGA, Provisional" data for each cancer type. Each data set was queried for RP coding mutations in any tumor sample. A literature search was performed in order to classify these observed mutations into three general categories: mutations in RPs previously implicated in a ribosomopathy, mutations identical to those previously identified in a ribosomopathy, and all other mutations. The literature search included the LOVD Diamond-Blackfan Anemia database (http://dbagenes.unito.it/home.php) as well as PubMed searches of each individual ribosomal protein gene identifier.

Quantification of rRNA processing

Total RNAs were purified using RNeasy columns (Qiagen, Inc. Valencia, CA) and then digested with TURBO-DNA *free* DNAse as recommended by the supplier (Thermo-Fisher, Pittsburgh, PA). RNA concentrations were determined with a Nanodrop ND-1000 instrument (NanoDrop Technologies Inc., Wilmington, DE, USA) and RNA integrity was evaluated with an Agilent 2100 Bioanalyzer (Agilent Technologies, Santa Clara,CA). RNA integrity number (RIN) values for all samples in the HCC mouse model and HB tumors >9 and those for HB control livers were >7.5.

To assess rRNA processing intermediates, we quantified 18S-ITS1, ITS1-5.8S, 5.8S-ITS2 and ITS2-18S junctions as depicted in Fig 2A using a Power SYBR® Green RNA-to-CT™ 1-Step Kit (Thermo-Fisher) on a StepOnePlus™ Real-Time PCR System (Thermo-Fisher). PCR primers (IDT, Inc., Coralville, IA) were selected from the murine 47S rRNA Gen Bank sequence BK000964.3 and comprised the following sequences:

18S-ITS1 (Fwd): 5’-AAGACGGTCGAACTTGACTATCTAG-3’ (nt 5797)

(54): 5’-GCCGCCGCTCCTCCACAGTCTC-3’ (nt 5900)

ITS1-5.8S (Fwd): 5’-CCCGTGAGTTCGCTCACACCCGA-3’ (nt 6844)

(54): 5’-CGCAGCTAGCTGCGTTCTTCATCGA-3’ (nt 6934)

5.8S-ITS2 (Fwd): 5’-TTGATCATCGACACTTCGAACGCAC-3’ (nt 6961)

(54): 5’-CGCAGCGGGTGACGCGATTGAT-3’ (nt 7059)

ITS2-28S (Fwd): 5’-CCTGAGACGGTTCGCCGGCTCGT-3’ (nt 8012)

(54): 5’-AGCGGGTCGCCACGTCTGATCTGA-3’ (nt 8153)

For each set of PCR primers, reactions were performed in triplicate on 5-8 liver or tumor samples. The results of each set of reactions were normalized to total 18S and 28S rRNA content, which were also obtained from triplicate reactions run in parallel. Control primers consisted of the following sets of oligonucleotides:

18S (Fwd): 5’-CTGAGAAACGGCTACCACATC-3’ (nt 4450)

(54): 5’- GCCTCGAAAGAGTCCTGTATTG-3’ (nt 4556)

28S (Fwd): 5’- GTAAACGGCGGGAGTAACTATG-3’ (nt 11522)

(54): 5’- GACAGTGGGAATCTCGTTCATC-3’ (nt 11619)

Each reaction (20 μL in a 96-well fast plate) consisted of 10 ng of RNA template, 0.16 μL RT Enzyme Mix, 10 μL the above-described RT-PCR Mix, 3.84 μL nuclease-free water and 1 μL of primer solution containing both forward and reverse primers at a concentration of 100 ng/μL each. Run conditions for RT-PCR reactions consisted of a 48°C hold for 30 minutes to catalyze reverse transcription followed by a 10-minute hold at 95°C. PCR conditions comprised 40 cycles of a 1 min 95°C melting period followed by a 1-minute 65°C annealing and extension period. Each sample was assayed in triplicate with variances seldom exceeding 5%. P-values were determined using Welch's t-test.

Tissue fractionation immuno-precipitation and immuno-fluorescent staining

All buffers were supplemented with standard protease and phosphatase inhibitors. Tissue was first washed with ice-cold PBS, minced into small pieces and then homogenized in cytoplasmic extraction buffer (200-250 mg tissue/2 ml of buffer). Cell breakage was monitored under a microscope. Homogenates were centrifuged at 500x g for 5 min at 4^0C and the resultant supernatants were used as the cytoplasmic fraction. Pellets were further subjected to membrane extraction buffer followed by centrifugation at 3000x g for 5 min at 4^0C to pellet nuclei. Nuclear pellets were divided into two halves. One half was used to solubilize nuclei and release chromatin-bound proteins by extracting with nuclear extraction buffer containing 5 mM $CaCl_2$. The other half was processed to isolate nucleoli as described (http://www.lamondlab.com). For this step, nuclear pellets were re-suspended in a buffer containing 10 mM Hepes, pH 7.9; 0.35 M sucrose and 0.5 mM $MgCl_2$ and sonicated on ice for 6 x 10 sec bursts with 10 sec cooling intervals. Sonicated lysates were layered over a buffer containing 10 mM Hepes, pH 7.9; 0.88 M sucrose and 0.5 mM $MgCl_2$ and centrifuged at 3000x g for 10 min at 4^0C. Nucleolar pellets thus

obtained were solubilized in a buffer containing Tris HCl, pH 8.0; 20 mM NaCl; 1 mM EDTA; 0.5% NP40 and 25 mM NaF. Fractions were analyzed by immuno-blotting for GAPDH (cytoplasmic marker), histone H3 (nuclear marker), fibrillarin (nuclear and nucleolar marker). Expression of p53, Mdm2, and p19^{ARF} was assessed by immuno-blotting across each of the fractions following SDS-PAGE.

For immuno-precipitations, freshly isolated cytoplasmic liver and tumor fractions were diluted to a final protein concentration of 3 mg/ml in "IP buffer" containing Tris HCl, pH 8.0; 20 mM NaCl; 1 mM EDTA; 0.5% NP40 and 25 mM NaF supplemented with protease and phosphatase inhibitors and subjected to two rounds of pre-clearing. The first round consisted of rocking 1 ml of lysate with 20 µl Protein G PLUS-agarose beads (Santa Cruz Biotechnology) for 1 h at 4^0C followed by centrifugation at 4000 rpm, 4^0C for 5 min, to remove agarose beads. The second round of clearing was performed following the addition of 20 µl of isotype specific IgG1-agarose conjugate (LifeSpan BioSciences inc., Seattle, WA) to the pre-cleared lysate for 1 h at 4^0C. Upon a brief centrifugation to again remove the agarose-conjugate, the fractions were equally divided. One portion was incubated with 20 µl mouse IgG1-agarose conjugated beads while the other part was incubated with Mdm2 antibody agarose-conjugated beads (Santa Cruz Biotechnology) overnight at 4^0C with gentle shaking. Beads were washed four times for 1 h each time at 4^0C with IP buffer to remove any unbound protein, followed by re-suspension in SDS sample buffer and denaturation at 95^0C for 4 min. Immunoprecipitates were further analyzed by gel electrophoresis and silver staining.

Immunofluorescent staining (Fig. 5C) was performed on liver and tumor frozen sections. Fresh tissues were first fixed in PBS-4% paraformaldehyde for 2-4 followed by an overnight incubation in PBS-40% sucrose at 4^0C. The fixed tissues were then embedded in Tissue Plus

O.C.T. Compound (SciGen Scientific, Gardenas, CA), frozen on dry ice and stored at -80^{0}C. Frozen tissues were cryo-sectioned, stained with antibodies against Mdm2 or p53 (Table S1) and counterstained with 4,6-Diamidino-2-phenylindole, dihydrochloride (DAPI) (1 μg/ml) for 5 min. Images were obtained on an Olympus Fluoview FV1000 confocal microscope. The nuclear: cytoplasmic distribution of Mdm2 and p53 were determined and quantified using the Particle Analysis program of Image J software (https://imagej.nih.gov/ij/).

Mass spectrometry

Briefly, excised gel bands were washed with HPLC water and de-stained exhaustively with 50% acetonitrile (ACN)/25 mM ammonium bicarbonate. Gel pieces were dehydrated with 100% ACN, reduced with 10 mM dithiothreitol (DTT) at 56^{0}C for 1 hour, followed by alkylation with 55 mM iodoacetamide (35) at room temperature for 45 min in the dark. Gel pieces were then again dehydrated with 100% ACN to remove excess DTT and IAA, and rehydrated with 20 ng/μl trypsin/25 mM ammonium bicarbonate and digested overnight at 37^{0}C. The resultant tryptic peptides were extracted with 70% ACN/5% formic acid, vacuum dried and re-constituted in 18 μl 0.1% formic acid.

Proteolytic peptides were analyzed by a nanoflow reverse-phased liquid chromatography tandem mass spectrometry (LC-MS/MS). They were loaded onto a C18 PicoChip^{TM} column packed with 10.5 cm of Reprosil C18 3 μm 120Å chromatography media with a 75 μm ID column and a 15 μm tip (New Objective, Inc., Woburn, MA) using a Dionex HPLC system (Dionex Ultimate 3000, ThermoFisher Scientific, San Jose, CA) operated with a double-split system (Dr. Steven Gygi from Department of Cell Biology, Harvard Medical School, personal communication) to provide an in-column nano-flow rate (~300 nl/min). Mobile phases used were

0.1% formic acid for A and 0.1% formic acid in acetonitrile for B. Peptides were eluted off the column using a 52 min gradient (2-40% B in 42 min, 40-95% B in 1min, 95% B for 1 min, 2% B for 8 min) and injected into a linear ion trap MS (LTQ-XL, ThermoFisher Scientific) through electrospray.

The MS instrument was operated in a date-dependent MS/MS mode in which each full MS spectrum was followed by MS/MS scans of the 5 most abundant molecular ions determined from full MS scan (acquired based on the setting of 1000 signal threshold, 10000 AGC target, 100 ms maximum accumulation time, 2.0 Da isolation width, 30 ms activation time and 35% normalized collision energy). Dynamic exclusion was enabled to minimize redundant selection of peptides previously selected for CID.

MS/MS spectra were searched using MASCOT search engine (Version 2.4.0, Matrix Science Ltd, London, UK) against the UniProt mouse proteome database. The following modifications were used: static modification of cysteine (carboxyamidomethylation, +57.05 Da), variable modification of methionine (oxidation, +15.99 Da). The mass tolerance was set at 1.4 Da for the precursor ions and 0.8 Da for the fragment ions. Peptide identifications were filtered using PeptideProphet™ and ProteinProphet® algorithms (LabKey, Inc. Seattle, WA) with a protein threshold cutoff of 99% and peptide threshold cutoff of 90% implemented in Scaffold™ (Proteome Software, Portland, Oregon, USA).

Figures

Figure A

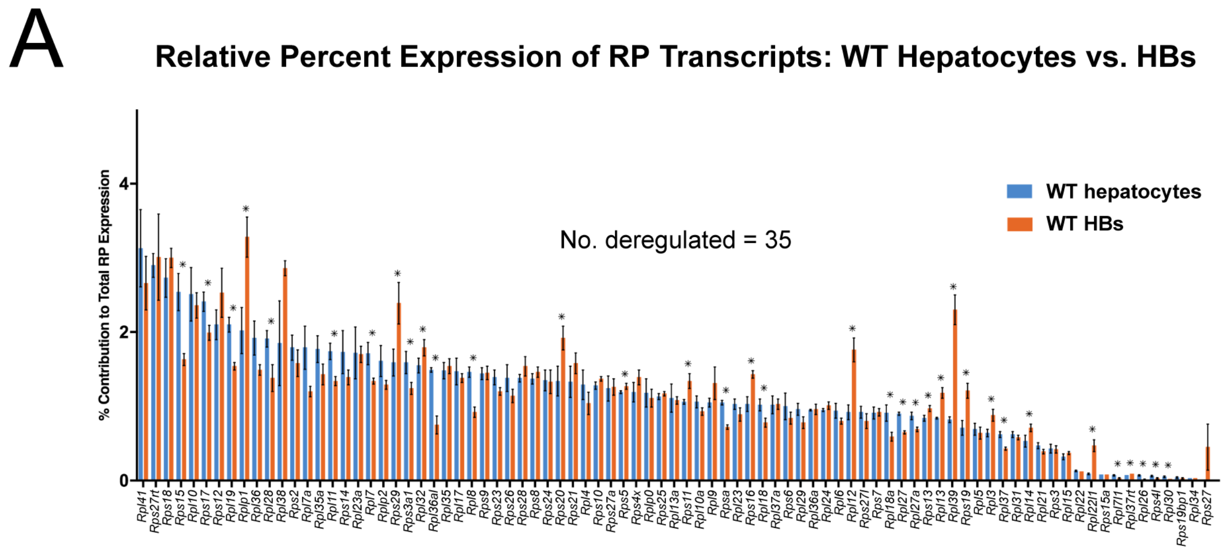

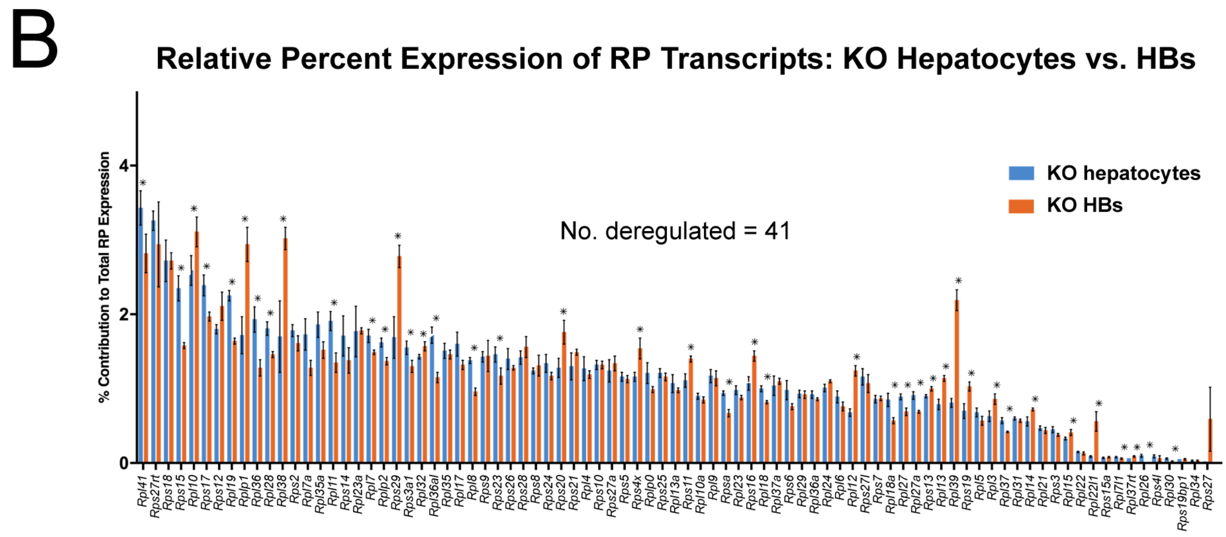

Figure B

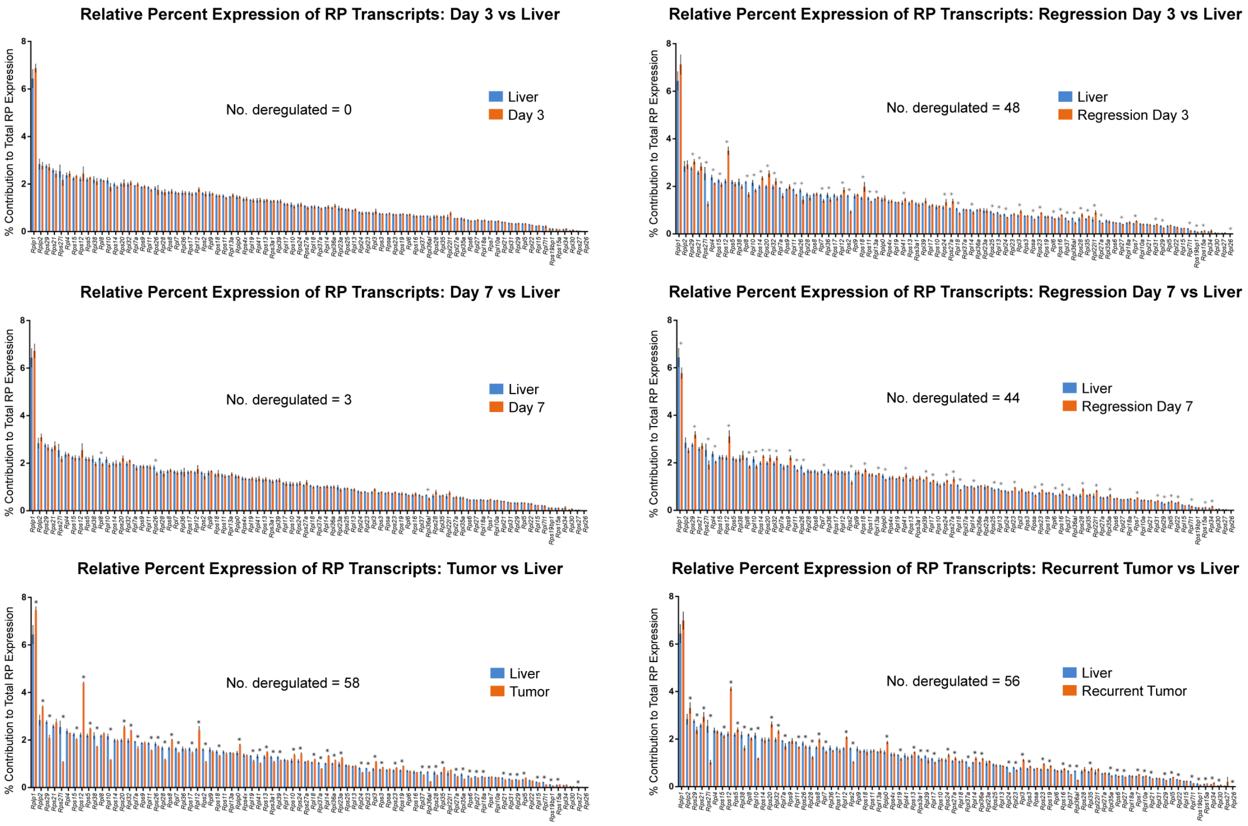

Relative Percent Expression of RP Transcripts: Day 3 vs Liver
% Contribution to Total RP Expression
No. deregulated = 0
Liver
Day 3
Relative Percent Expression of RP Transcripts: Regression Day 3 vs Liver
% Contribution to Total RP Expression
No. deregulated = 48
Liver
Regression Day 3
Relative Percent Expression of RP Transcripts: Day 7 vs Liver
% Contribution to Total RP Expression
No. deregulated = 3
Liver
Day 7
Relative Percent Expression of RP Transcripts: Regression Day 7 vs Liver
% Contribution to Total RP Expression
No. deregulated = 44
Liver
Regression Day 7
Relative Percent Expression of RP Transcripts: Tumor vs Liver
% Contribution to Total RP Expression
No. deregulated = 58
Liver
Tumor
Relative Percent Expression of RP Transcripts: Recurrent Tumor vs Liver
% Contribution to Total RP Expression
No. deregulated = 56
Liver
Recurrent Tumor

Figure C

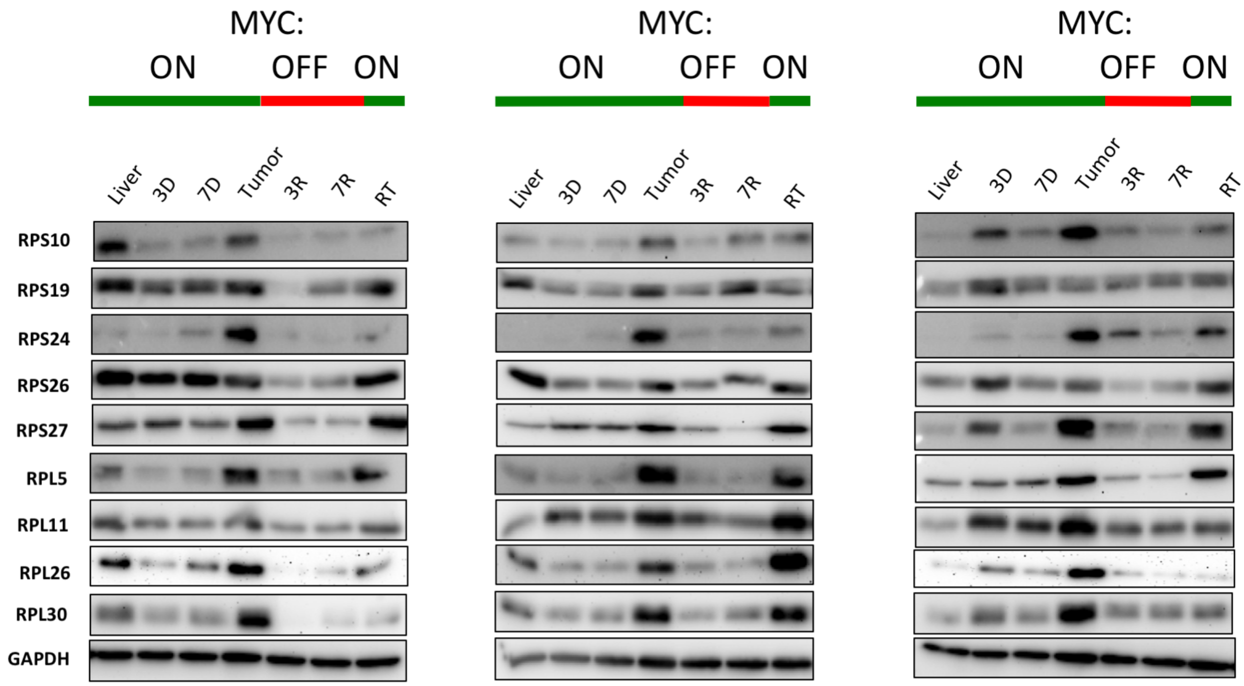
MYC:
ON
OFF
ON
Liver
3D
7D
Tumor
3R
7R
RT
RPS10
RPS19
RPS24
RPS26
RPS27
RPL5
RPL11
RPL26
RPL30
GAPDH

Figure D

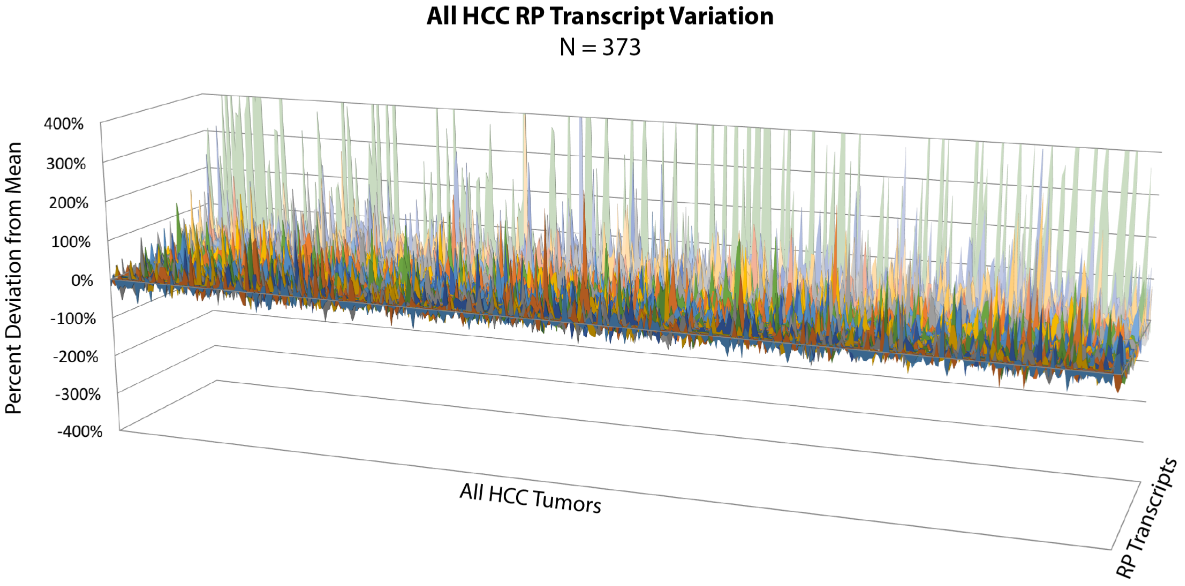
All HCC RP Transcript Variation
N = 373
Percent Deviation from Mean
400%
300%
200%
100%
0%
-100%
-200%
-300%
-400%
All HCC Tumors
RP Transcripts

Figure E

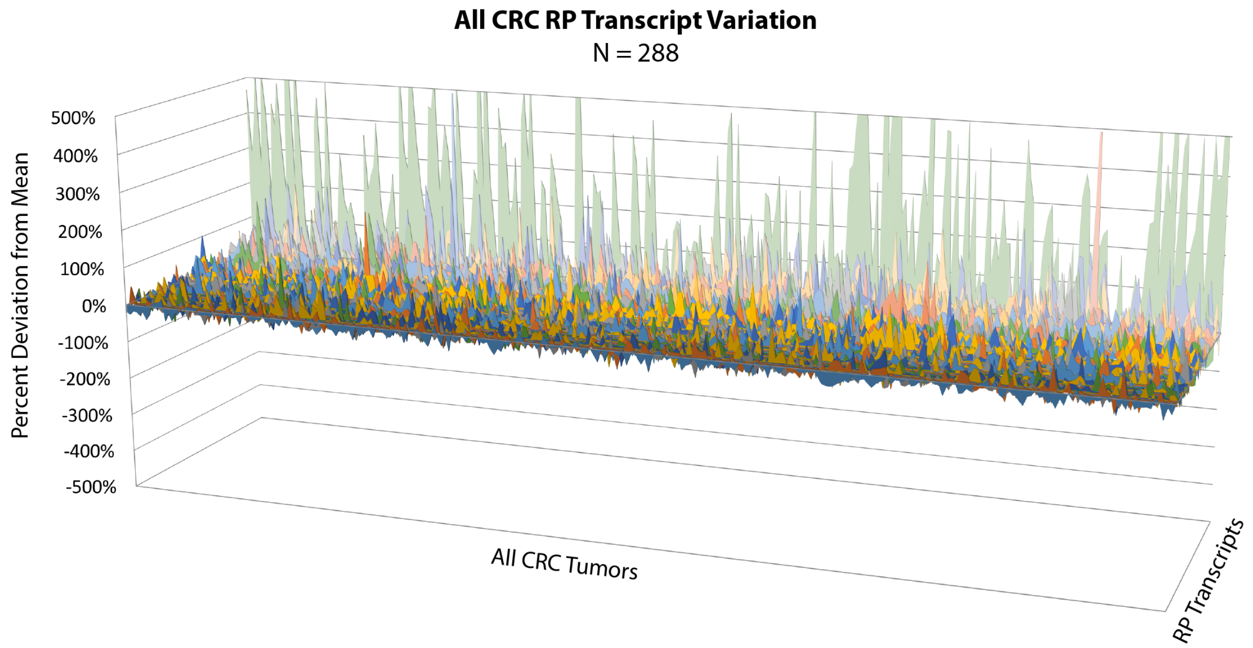

All CRC RP Transcript Variation
N = 288
500%
400%
300%
200%
100%
0%
-100%
-200%
-300%
-400%
-500%
Percent Deviation from Mean
All CRC Tumors
RP Transcripts

Figure F

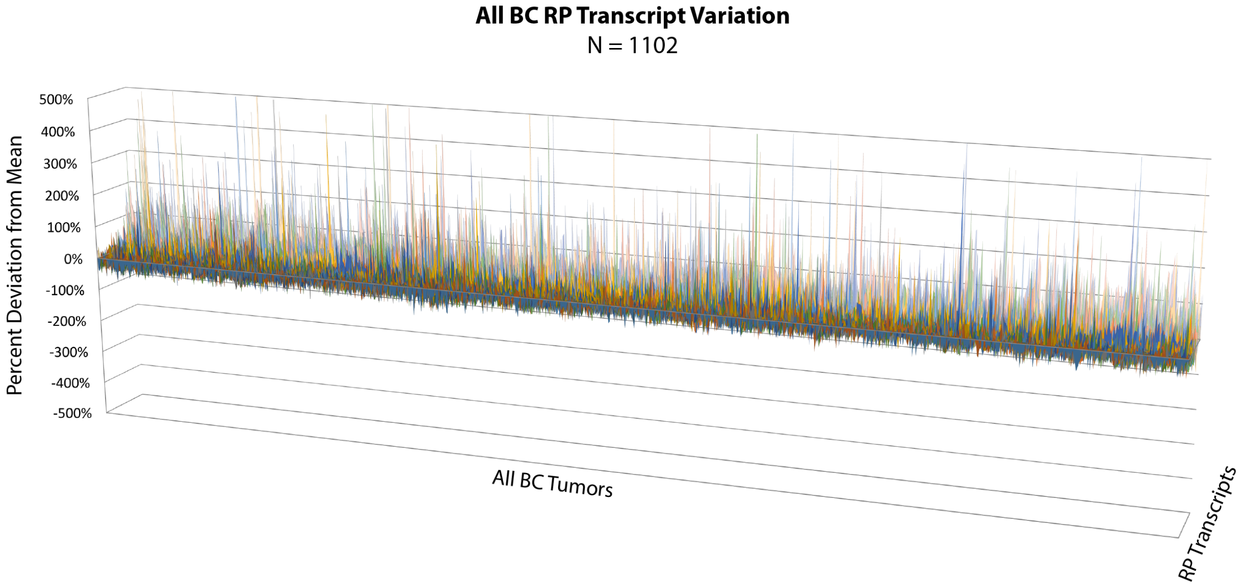

All BC RP Transcript Variation
N = 1102
500%
400%
300%
200%
100%
0%
-100%
-200%
-300%
-400%
-500%
Percent Deviation from Mean
All BC Tumors
RP Transcripts

Figure G

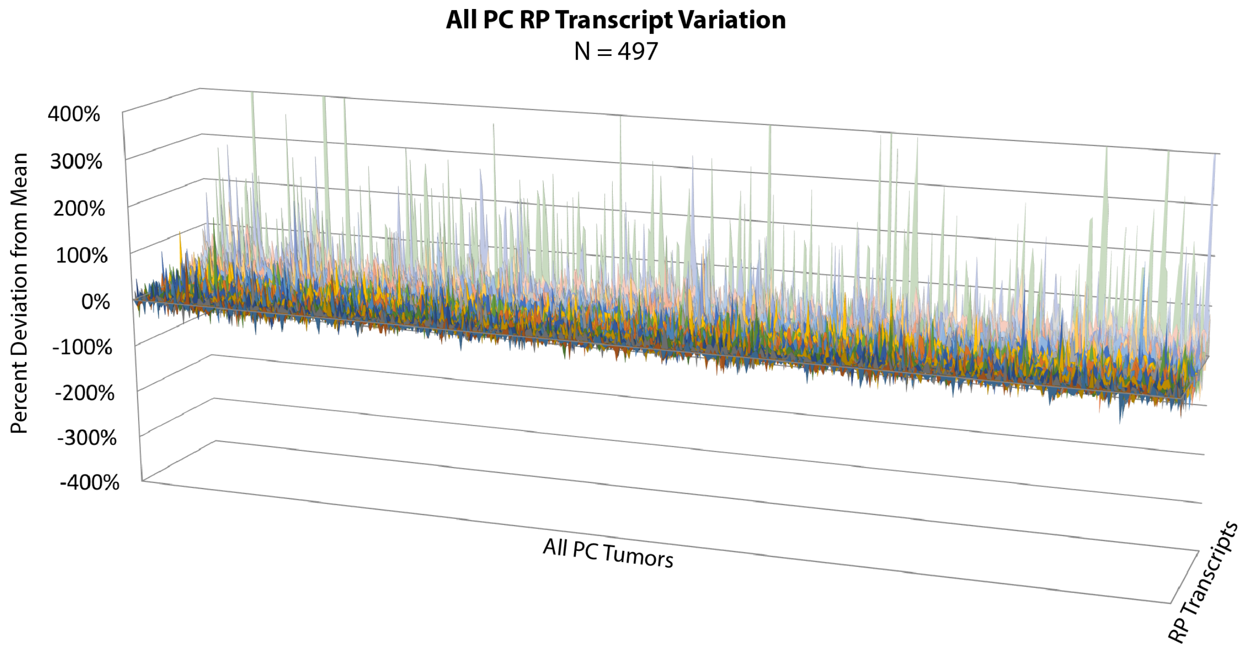
All PC RP Transcript Variation
N = 497
400%
300%
200%
100%
0%
-100%
-200%
-300%
-400%
Percent Deviation from Mean
All PC Tumors
RP Transcripts

Figure H

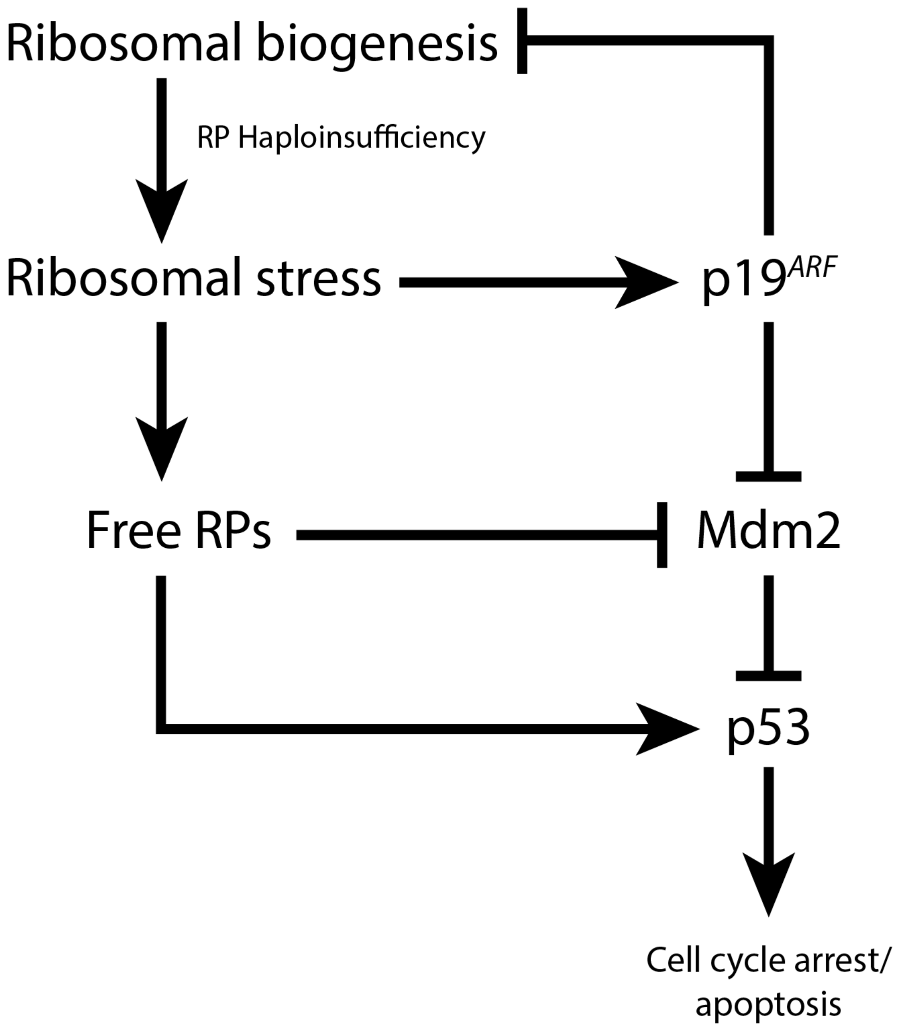

Ribosomal biogenesis
RP Haploinsufficiency
Ribosomal stress
p19ARF
Free RPs
Mdm2
p53
Cell cycle arrest/
apoptosis

Figure I

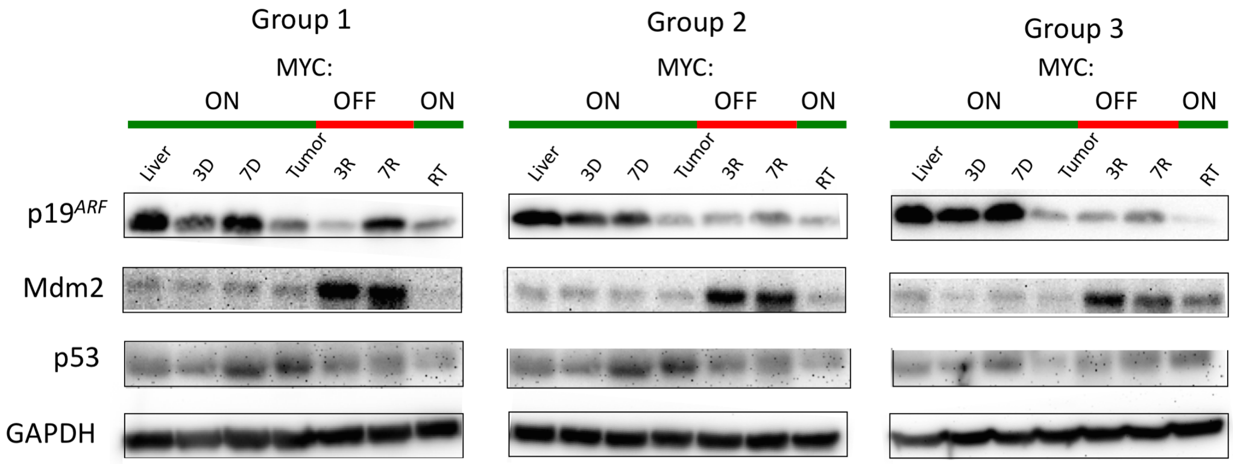
Group 1
Group 2
Group 3
MYC:
ON
OFF
ON
Liver
3D
7D
Tumor
3R
7R
RT
p19ARF
Mdm2
p53
GAPDH

Figure J

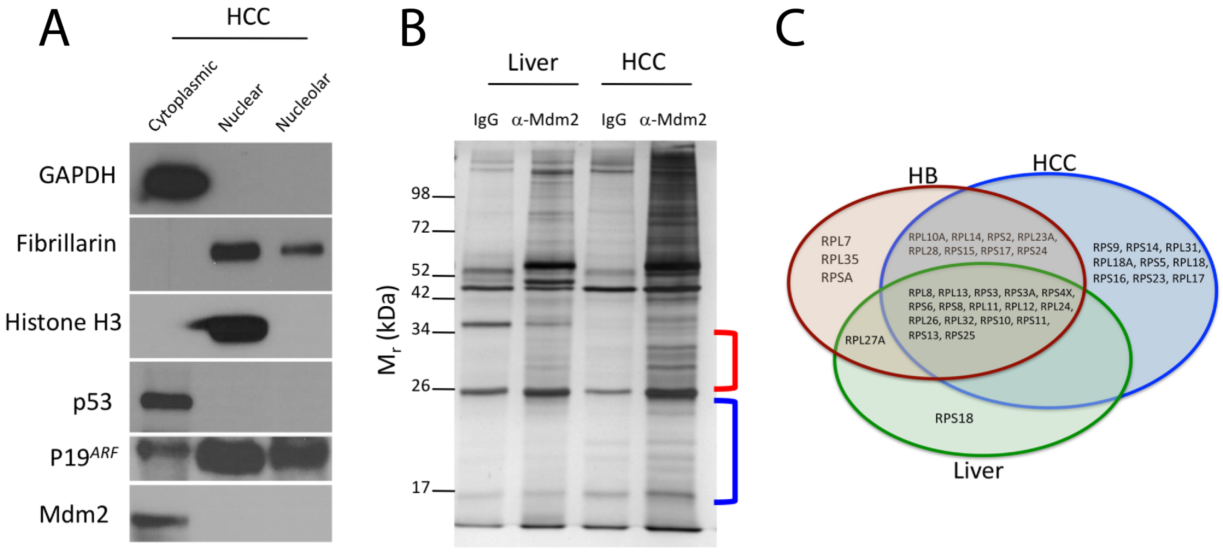
A
HCC
Cytoplasmic
Nuclear
Nucleolar
GAPDH
Fibrillarin
Histone H3
p53
P19ARF
Mdm2
B
Liver
HCC
IgG
α-Mdm2
IgG
α-Mdm2
Mr (kDa)
98
72
52
42
34
26
17
C
HB
HCC
Liver
RPL7
RPL35
RPSA
RPL10A, RPL14, RPS2, RPL23A,
RPL28, RPS15, RPS17, RPS24
RPS9, RPS14, RPL31,
RPL18A, RPS5, RPL18,
RPS16, RPS23, RPL17
RPL8, RPL13, RPS3, RPS3A, RPS4X,
RPS6, RPS8, RPL11, RPL12, RPL24,
RPL26, RPL32, RPS10, RPS11,
RPS13, RPS25
RPL27A
RPS18

Figure K

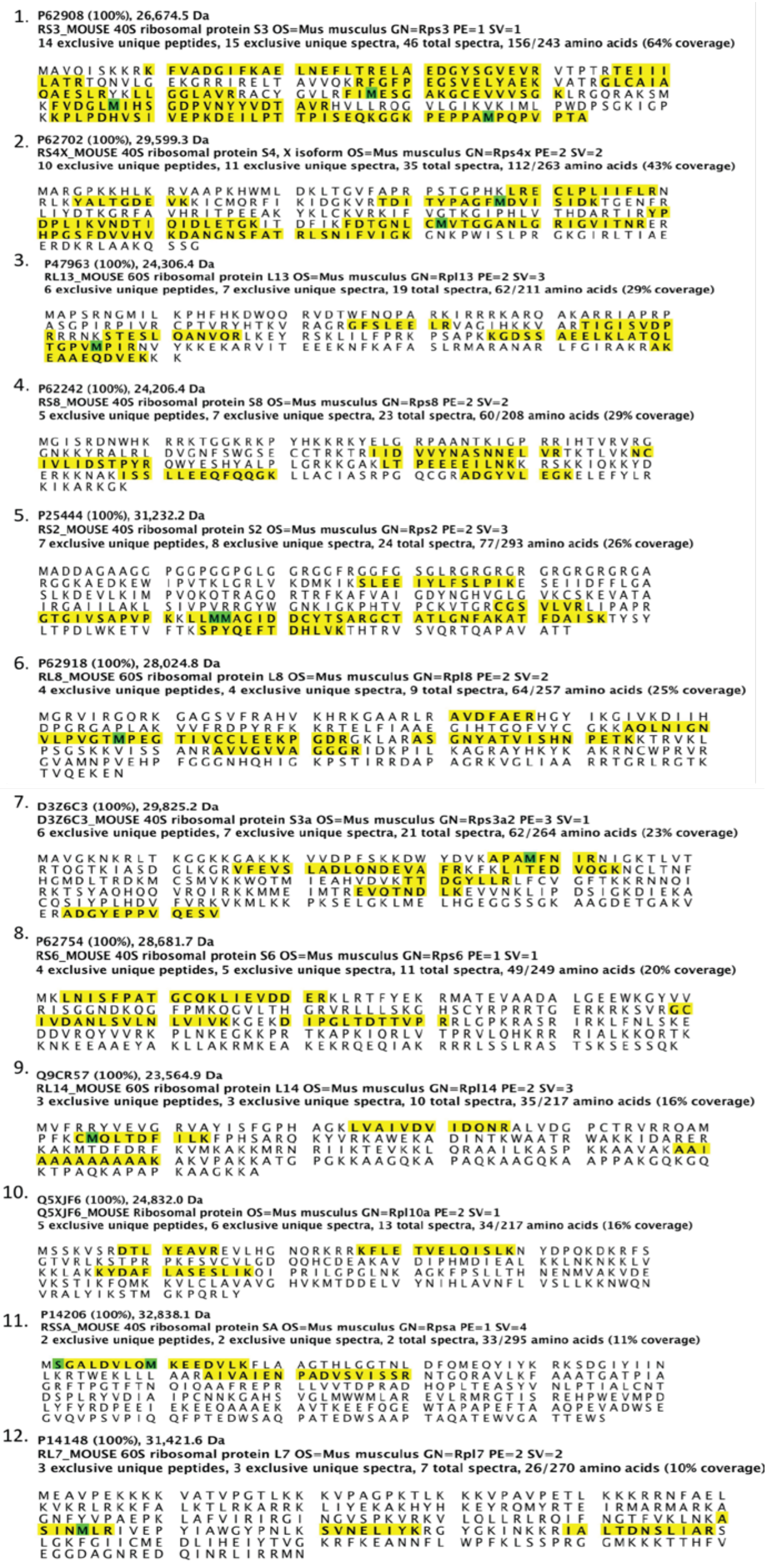
1. P62908 (100%), 26,674.5 Da
RS3_MOUSE 40S ribosomal protein S3 OS=Mus musculus GN=Rps3 PE=1 SV=1
14 exclusive unique peptides, 15 exclusive unique spectra, 46 total spectra, 156/243 amino acids (64% coverage)
2. P62702 (100%), 29,599.3 Da
RS4X_MOUSE 40S ribosomal protein S4, X isoform OS=Mus musculus GN=Rps4x PE=2 SV=2
10 exclusive unique peptides, 11 exclusive unique spectra, 35 total spectra, 112/263 amino acids (43% coverage)
3. P47963 (100%), 24,306.4 Da
RL13_MOUSE 60S ribosomal protein L13 OS=Mus musculus GN=Rpl13 PE=2 SV=3
6 exclusive unique peptides, 7 exclusive unique spectra, 19 total spectra, 62/211 amino acids (29% coverage)
4. P62242 (100%), 24,206.4 Da
RS8_MOUSE 40S ribosomal protein S8 OS=Mus musculus GN=Rps8 PE=2 SV=2
5 exclusive unique peptides, 7 exclusive unique spectra, 23 total spectra, 60/208 amino acids (29% coverage)
5. P25444 (100%), 31,232.2 Da
RS2_MOUSE 40S ribosomal protein S2 OS=Mus musculus GN=Rps2 PE=2 SV=3
7 exclusive unique peptides, 8 exclusive unique spectra, 24 total spectra, 77/293 amino acids (26% coverage)
6. P62918 (100%), 28,024.8 Da
RL8_MOUSE 60S ribosomal protein L8 OS=Mus musculus GN=Rpl8 PE=2 SV=2
4 exclusive unique peptides, 4 exclusive unique spectra, 9 total spectra, 64/257 amino acids (25% coverage)
7. D3Z6C3 (100%), 29,825.2 Da
D3Z6C3_MOUSE 40S ribosomal protein S3a OS=Mus musculus GN=Rps3a2 PE=3 SV=1
6 exclusive unique peptides, 7 exclusive unique spectra, 21 total spectra, 62/264 amino acids (23% coverage)
8. P62754 (100%), 28,681.7 Da
RS6_MOUSE 40S ribosomal protein S6 OS=Mus musculus GN=Rps6 PE=1 SV=1
4 exclusive unique peptides, 5 exclusive unique spectra, 11 total spectra, 49/249 amino acids (20% coverage)
9. Q9CR57 (100%), 23,564.9 Da
RL14_MOUSE 60S ribosomal protein L14 OS=Mus musculus GN=Rpl14 PE=2 SV=3
3 exclusive unique peptides, 3 exclusive unique spectra, 10 total spectra, 35/217 amino acids (16% coverage)
10. Q5XJF6 (100%), 24,832.0 Da
Q5XJF6_MOUSE Ribosomal protein OS=Mus musculus GN=Rpl10a PE=2 SV=1
5 exclusive unique peptides, 6 exclusive unique spectra, 13 total spectra, 34/217 amino acids (16% coverage)
11. P14206 (100%), 32,838.1 Da
RSSA_MOUSE 40S ribosomal protein SA OS=Mus musculus GN=Rpsa PE=1 SV=4
2 exclusive unique peptides, 2 exclusive unique spectra, 2 total spectra, 33/295 amino acids (11% coverage)
12. P14148 (100%), 31,421.6 Da
RL7_MOUSE 60S ribosomal protein L7 OS=Mus musculus GN=Rpl7 PE=2 SV=2
3 exclusive unique peptides, 3 exclusive unique spectra, 7 total spectra, 26/270 amino acids (10% coverage)

Figure L

1. **P62908 (100%), 26,674.5 Da**
RS3_MOUSE 40S ribosomal protein S3 OS=Mus musculus GN=Rps3 PE=1 SV=1
8 exclusive unique peptides, 9 exclusive unique spectra, 16 total spectra, 94/243 amino acids (39% coverage)

```
MAVOISKKRK FVADGIFKAE LNEFLTRELA EDGYSGVEVR VTPTRTEIII
LATRTONVLG EKGRRIRELT AVVOKRFGFP EGSVELYAEK VATRGLCAIA
OAESLRYKLL GGLAVRRACY GVLRFIMESG AKGCEVVVSG KLRGORAKSM
KFVDGLMIHS GDPVNYYVDT AVRHVLLROG VLGIKVKIML PWDPSGKIGP
KKPLPDHVSI VEPKDEILPT TPISEQKGGK PEPPAMPQPV PTA
```

2. **P62242 (100%), 24,206.4 Da**
RS8_MOUSE 40S ribosomal protein S8 OS=Mus musculus GN=Rps8 PE=2 SV=2
5 exclusive unique peptides, 5 exclusive unique spectra, 8 total spectra, 60/208 amino acids (29% coverage)

```
MGISRDNWHK RRKTGGKRKP YHKKRKYELG RPAANTKIGP RRIHTVRVRG
GNKKYRALRL DVGNFSWGSE CCTRKTRIID VVYNASNNEL VRTKTLVKNC
IVLIDSTPYR OWYESHYALP LGRKKGAKLT PEEEEILNKK RSKKIOKKYD
ERKKNAKISS LLEEQFQQGK LLACIASRPG QCGRADGYVL EGKELEFYLR
KIKARKGK
```

3. **P62702 (100%), 29,599.3 Da**
RS4X_MOUSE 40S ribosomal protein S4, X isoform OS=Mus musculus GN=Rps4x PE=2 SV=2
5 exclusive unique peptides, 7 exclusive unique spectra, 12 total spectra, 66/263 amino acids (25% coverage)

```
MARGPKKHLK RVAAPKHWML DKLTGVFAPR PSTGPHKLRE CLPLIIFLRN
RLKYALTGDE VKKICMORFI KIDGKVRTDI TYPAGFMDVI SIDKTGENFR
LIYDTKGRFA VHRITPEEAK YKLCKVRKIF VGTKGIPHLV THDARTIRYP
DPLIKVNDTI OIDLETGKIT DFIKFDTGNL CMVTGGANLG RIGVITNRER
HPGSFDVVHV KDANGNSFAT RLSNIFVIGK GNKPWISLPR GKGIRLTIAE
ERDKRLAAKQ SSG
```

4. **P62754 (100%), 28,681.7 Da**
RS6_MOUSE 40S ribosomal protein S6 OS=Mus musculus GN=Rps6 PE=1 SV=1
3 exclusive unique peptides, 3 exclusive unique spectra, 3 total spectra, 32/249 amino acids (13% coverage)

```
MKLNISFPAT GCQKLIEVDD ERKLRTFYEK RMATEVAADA LGEEWKGYVV
RISGGNDKOG FPMKOGVLTH GRVRLLLSKG HSCYRPRRTG ERKRKSVRGC
IVDANLSVLN LVIVKKGEKD IPGLTDTTVP RRLGPKRASR IRKLFNLSKE
DDVROYVVRK PLNKEGKKPR TKAPKIORLV TPRVLOHKRR RIALKKORTK
KNKEEAAEYA KLLAKRMKEA KEKRQEQIAK RRRLSSLRAS TSKSESSQK
```

5. **P97351 (100%), 29,885.3 Da**
RS3A_MOUSE 40S ribosomal protein S3a OS=Mus musculus GN=Rps3a PE=1 SV=3
3 exclusive unique peptides, 3 exclusive unique spectra, 3 total spectra, 34/264 amino acids (13% coverage)

```
MAVGKNKRLT KGGKKGAKKK VVDPFSKKDW YDVKAPAMFN IRNIGKTLVT
RTOGTKIASD GLKGRVFEVS LADLONDEVA FRKFKLITED VOGKNCLTNF
HGMDLTRDKM CSMVKKWOTM IEAHVDVKTT DGYLLRLFCV GFTKKRNNOI
RKTSYAOHOO VROIRKKMME IMTREVOTND LKEVVNKLIP DSIGKDIEKA
COSIYPLHDV FVRKVKMLKK PKFELGKLME LHGEGGSSGK AAGDETGAKV
ERADGYEPPV QESV
```

6. **P47963 (100%), 24,306.4 Da**
RL13_MOUSE 60S ribosomal protein L13 OS=Mus musculus GN=Rpl13 PE=2 SV=3
2 exclusive unique peptides, 2 exclusive unique spectra, 2 total spectra, 19/211 amino acids (9% coverage)

```
MAPSRNGMIL KPHFHKDWOO RVDTWFNOPA RKIRRRKARO AKARRIAPRP
ASGPIRPIVR CPTVRYHTKV RAGRGFSLEE LRVAGIHKKV ARTIGISVDP
RRRNKSTESL OANVORLKEY RSKLILFPRK PSAPKKGDSS AEELKLATOL
TGPVMPIRNV YKKEKARVIT EEEKNFKAFA SLRMARANAR LFGIRAKRAK
EAAEQDVEKK K
```

7. **P62918 (100%), 28,024.8 Da**
RL8_MOUSE 60S ribosomal protein L8 OS=Mus musculus GN=Rpl8 PE=2 SV=2
2 exclusive unique peptides, 2 exclusive unique spectra, 2 total spectra, 18/257 amino acids (7% coverage)

```
MGRVIRGORK GAGSVFRAHV KHRKGAARLR AVDFAERHGY IKGIVKDIIH
DPGRGAPLAK VVFRDPYRFK KRTELFIAAE GIHTGOFVYC GKKAOLNIGN
VLPVGTMPEG TIVCCLEEKP GDRGKLARAS GNYATVISHN PETKKTRVKL
PSGSKKVISS ANRAVVGVVA GGGRIDKPIL KAGRAYHKYK AKRNCWPRVR
GVAMNPVEHP FGGGNHQHIG KPSTIRRDAP AGRKVGLIAA RRTGRLRGTK
TVQEKEN
```

Figure M

1. P35979 (100%), 17,805.0 Da
RL12_MOUSE 60S ribosomal protein L12 OS=Mus musculus GN=Rpl12 PE=1 SV=2
7 exclusive unique peptides, 9 exclusive unique spectra, 25 total spectra, 98/165 amino acids (59% coverage)

```
MPPKFDPNEV KVVYLRCTGG EVGATSALAP KIGPLGLSPK KVGDDIAKAT
GDWKGLRITV KLTIQNRQAQ IEVVPSASAL IIKALKEPPR DRKKQKNIKH
SGNITFDEIV NIARQMRHRS LARELSGTIK EILGTAQSVG CNVDGRHPHD
IIDDINSGAV ECPAS
```

2. P63276 (100%), 15,524.5 Da
RS17_MOUSE 40S ribosomal protein S17 OS=Mus musculus GN=Rps17 PE=1 SV=2
5 exclusive unique peptides, 6 exclusive unique spectra, 11 total spectra, 66/135 amino acids (49% coverage)

```
MGRVRTKTVK KAARVIIEKY YTRLGNDFHT NKRVCEEIAI IPSKKLRNKI
AGYVTHLMKR IQRGPVRGIS IKLQEEERER RDNYVPEVSA LDQEIIEVDP
DTKEMLKLLD FGSLSNLQVT QPTVGMNFKT PRGAV
```

3. P62301 (100%), 17,223.3 Da
RS13_MOUSE 40S ribosomal protein S13 OS=Mus musculus GN=Rps13 PE=2 SV=2
7 exclusive unique peptides, 7 exclusive unique spectra, 17 total spectra, 65/151 amino acids (43% coverage)

```
MGRMHAPGKG LSQSALPYRR SVPTWLKLTS DDVKEQIYKL AKKGLTPSQI
GVILRDSHGV AQVRFVTGNK ILRILKSKGL APDLPEDLYH LIKKAVAVRK
HLERNRKDKD AKFRLILIES RIHRLARYYK TKRVLPPNWK YESSTASALV
A
```

4. P62281 (100%), 18,431.3 Da
RS11_MOUSE 40S ribosomal protein S11 OS=Mus musculus GN=Rps11 PE=2 SV=3
8 exclusive unique peptides, 8 exclusive unique spectra, 19 total spectra, 64/158 amino acids (41% coverage)

```
MADIQTERAY QKQPTIFQNK KRVLLGETGK EKLPRYYKNI GLGFKTPKEA
IEGTYIDKKC PFTGNVSIRG RILSGVVTKM KMQRTIVIRR DYLHYIRKYN
RFEKRHKNMS VHLSPCFRDV QIGDIVTVGE CRPLSKTVRF NVLKVTKAAG
TKKQFQKF
```

5. P62849 (100%), 15,423.8 Da
RS24_MOUSE 40S ribosomal protein S24 OS=Mus musculus GN=Rps24 PE=2 SV=1
4 exclusive unique peptides, 5 exclusive unique spectra, 8 total spectra, 46/133 amino acids (35% coverage)

```
MNDTVTIRTR KFMTNRLLQR KQMVIDVLHP GKATVPKTEI REKLAKMYKT
TPDVIFVFGF RTHFGGGKTT GFGMIYDSLD YAKKNEPKHR LARHGLYEKK
KTSRKQRKER KNRMKKVRGT AKANVGAGKK PKE
```

6. Q9CXW4 (100%), 20,253.2 Da
RL11_MOUSE 60S ribosomal protein L11 OS=Mus musculus GN=Rpl11 PE=1 SV=4
5 exclusive unique peptides, 5 exclusive unique spectra, 13 total spectra, 55/178 amino acids (31% coverage)

```
MAQDQGEKEN PMRELRIRKL CLNICVGESG DRLTRAAKVL EQLTGQTPVF
SKARYTVRSF GIRRNEKIAV HCTVRGAKAE EILEKGLKVR EYELRKNNFS
DTGNFGFGIQ EHIDLGIKYD PSIGIYGLDF YVVLGRPGFS IADKKRRTGC
IGAKHRISKE EAMRWFQQKY DGIILPGK
```

7. P62751 (100%), 17,696.2 Da
RL23A_MOUSE 60S ribosomal protein L23a OS=Mus musculus GN=Rpl23a PE=1 SV=1
5 exclusive unique peptides, 5 exclusive unique spectra, 17 total spectra, 46/156 amino acids (29% coverage)

```
MAPKAKKEAP APPKAEAKAK ALKAKKAVLK GVHSHKKKKI RTSPTFRRPK
TLRLRRQPKY PRKSAPRRNK LDHYAIIKFP LTTESAMKKI EDNNTLVFIV
DVKANKHQIK QAVKKLYDID VAKVNTLIRP DGEKKAYVRL APDYDALDVA
NKIGII
```

8. P47963 (100%), 24,306.4 Da
RL13_MOUSE 60S ribosomal protein L13 OS=Mus musculus GN=Rpl13 PE=2 SV=3
6 exclusive unique peptides, 7 exclusive unique spectra, 19 total spectra, 62/211 amino acids (29% coverage)

```
MAPSRNGMIL KPHFHKDWQQ RVDTWFNQPA RKIRRRKARQ AKARRIAPRP
ASGPIRPIVR CPTVRYHTKV RAGRGFSLEE LRVAGIHKKV ARTIGISVDP
RRRNKSTESL QANVQRLKEY RSKLILFPRK PSAPKKGDSS AEELKLATQL
TGPVMPIRNV YKKEKARVIT EEEKNFKAFA SLRMARANAR LFGIRAKRAK
EAAEQDVEKK K
```

9. P62911 (100%), 15,860.4 Da
RL32_MOUSE 60S ribosomal protein L32 OS=Mus musculus GN=Rpl32 PE=2 SV=2
3 exclusive unique peptides, 3 exclusive unique spectra, 10 total spectra, 37/135 amino acids (27% coverage)

```
MAALRPLVKP KIVKKRTKKF IRHQSDRYVK IKRNWRKPRG IDNRVRRRFK
GQILMPNIGY GSNKKTKHML PSGFRKFLVH NVKELEVLLM CNKSYCAEIA
HNVSSKNRKA IVERAAQLAI RVTNPNARLR SEENE
```

10. P62852 (100%), 13,743.0 Da
RS25_MOUSE 40S ribosomal protein S25 OS=Mus musculus GN=Rps25 PE=2 SV=1
4 exclusive unique peptides, 4 exclusive unique spectra, 17 total spectra, 30/125 amino acids (24% coverage)

```
MPPKDDKKKK DAGKSAKKDK DPVNKSGGKA KKKKWSKGKV RDKLNNLVLF
DKATYDKLCK EVPNYKLITP AVVSERLKIR GSLARAALQE LLSKGLIKLV
SKHRAQVIYT RNTKGGDAPA AGEDA
```

11. P62843 (100%), 17,040.8 Da
RS15_MOUSE 40S ribosomal protein S15 OS=Mus musculus GN=Rps15 PE=2 SV=2
2 exclusive unique peptides, 3 exclusive unique spectra, 8 total spectra, 32/145 amino acids (22% coverage)

```
MAEVEQKKKR TFRKFTYRGV DLDQLLDMSY EQLMQLYSAR QRRRLNRGLR
RKQHSLLKRL RKAKKEAPPM EKPEVVKTHL RDMIILPEMV GSMVGVYNGK
TFNQVEIKPE MIGHYLGEFS ITYKPVKHGR PGIGATHSSR FIPLK
```

12. Q6ZWV7 (100%), 14,553.6 Da
RL35_MOUSE 60S ribosomal protein L35 OS=Mus musculus GN=Rpl35 PE=2 SV=1
2 exclusive unique peptides, 2 exclusive unique spectra, 8 total spectra, 23/123 amino acids (19% coverage)

```
MAKIKARDLR GKKKEELLKQ LDDLKVELSQ LRVAKVTGGA ASKLSKIRVV
RKSIARVLTV INQTQKENLR KFYKGKKYKP LDLRPKKTRA MRRRLTKHEE
KLKTKKQQRK ERLYPLRKYA VKA
```

13. P61255 (100%), 17,258.4 Da
RL26_MOUSE 60S ribosomal protein L26 OS=Mus musculus GN=Rpl26 PE=2 SV=1
4 exclusive unique peptides, 4 exclusive unique spectra, 18 total spectra, 26/145 amino acids (18% coverage)

```
MKFNPFVTSD RSKNRKRHFN APSHIRRKIM SSPLSKELRQ KYNVRSMPIR
KDDEVQVVRG HYKGQQIGKV VQVYRKKYVI YIERVQREKA NGTTVHVGIH
PSKVVITRLK LDKDRKKILE RKAKSRQVGK EKGKYKEETI EKMQE
```

14. P41105 (100%), 15,733.7 Da
RL28_MOUSE 60S ribosomal protein L28 OS=Mus musculus GN=Rpl28 PE=1 SV=2
2 exclusive unique peptides, 2 exclusive unique spectra, 7 total spectra, 22/137 amino acids (16% coverage)

```
MSAHLQWMVV RNCSSFLIKR NKQTYSTEPN NLKARNSFRY NGLIHRKTVG
VEPAADGKGV VVVMKRRSGQ RKPATSYVRT TINKNARATL SSIRHMIRKN
KYRPDLRMAA IRRASAILRS QKPVVVKRKR TRPTKSS
```

15. P14115 (100%), 16,605.4 Da
RL27A_MOUSE 60S ribosomal protein L27a OS=Mus musculus GN=Rpl27a PE=2 SV=5
2 exclusive unique peptides, 2 exclusive unique spectra, 11 total spectra, 23/148 amino acids (16% coverage)

```
MPSRLRKTRK LRGHVSHGHG RIGKHRKHPG GRGNAGGMHH HRINFDKYHP
GYFGKVGMRH YHLKRNQSFC PTVNLDKLWT LVSEQTRVNA AKNKTGVAPI
IDVVRSGYYK VLGKGKLPKQ PVIVKAKFFS RRAEEKIKGV GGACVLVA
```

16. P63325 (100%), 18,916.3 Da
RS10_MOUSE 40S ribosomal protein S10 OS=Mus musculus GN=Rps10 PE=1 SV=1
2 exclusive unique peptides, 2 exclusive unique spectra, 6 total spectra, 23/165 amino acids (14% coverage)

```
MLMPKKNRIA IYELLFKEGV MVAKKDVHMP KHPELADKNV PNLHVMKAMQ
SLKSRGYVKE QFAWRHFYWY LTNEGIQYLR DYLHLPPEIV PATLRRSRPE
TGRPRPKGPE GERPARFTRG EADRDTYRRS AVPPGADKKA EAGAGSATEF
QFRGGFGRGR GQPPQ
```

17. Q8BP67 (100%), 17,779.5 Da
RL24_MOUSE 60S ribosomal protein L24 OS=Mus musculus GN=Rpl24 PE=2 SV=2
2 exclusive unique peptides, 3 exclusive unique spectra, 11 total spectra, 21/157 amino acids (13% coverage)

```
MKVELCSFSG YKIYPGHGRR YARTDGKVFQ FLNAKCESAF LSKRNPRQIN
WTVLYRRKHK KGQSEEIQKK RTRRAVKFQR AITGASLADI MAKRNQKPEV
RKAQREQAIR AAKEAKKAKQ ASKKTAMAAA KAPTKAAPKQ KIVKPVKVSA
PRVGGKR
```

Figure N

1. P62270 (100%), 17,719.3 Da
RS18_MOUSE 40S ribosomal protein S18 OS=Mus musculus GN=Rps18 PE=1 SV=3
6 exclusive unique peptides, 6 exclusive unique spectra, 7 total spectra, 58/152 amino acids (38% coverage)

MSLVIPEKFQ HILRVLNTNI DGRRKIAFAI TAIKGVGRRY AHVVLRKADI
DLTKRAGELT EDEVERVITI MONPROYKIP DWFLNROKDV KDGKYSOVLA
NGLDNKLRED LERLKKIRAH RGLRHFWGLR VRGQHTKTTG RRGRTVGVSK
KK

2. P35979 (100%), 17,805.0 Da
RL12_MOUSE 60S ribosomal protein L12 OS=Mus musculus GN=Rpl12 PE=1 SV=2
4 exclusive unique peptides, 4 exclusive unique spectra, 4 total spectra, 48/165 amino acids (29% coverage)

MPPKFDPNEV KVVYLRCTGG EVGATSALAP KIGPLGLSPK KVGDDIAKAT
GDWKGLRITV KLTIONROAO IEVVPSASAL IIKALKEPPR DRKKOKNIKH
SGNITFDEIV NIAROMRHRS LARELSGTIK EILGTAQSVG CNVDGRHPHD
IIDDINSGAV ECPAS

3. E9Q132 (100%), 15,347.5 Da
E9Q132_MOUSE 60S ribosomal protein L24 OS=Mus musculus GN=Rpl24 PE=2 SV=1
4 exclusive unique peptides, 5 exclusive unique spectra, 5 total spectra, 30/133 amino acids (23% coverage)

MKVELCSFSG YKIYPGHGRR YARTDGKVFO FLNAKCESAF LSKRNPROIN
WTVLYRRKHK KGOSEEIOKK RTRRAVKFOR AITGASLADI MAKRNQKPEV
RKAQREQAIR AAKEAKKAKQ ASKKTAMAAA KVY

4. Q9CXW4 (100%), 20,253.2 Da
RL11_MOUSE 60S ribosomal protein L11 OS=Mus musculus GN=Rpl11 PE=1 SV=4
3 exclusive unique peptides, 3 exclusive unique spectra, 3 total spectra, 39/178 amino acids (22% coverage)

MAODOGEKEN PMRELRIRKL CLNICVGESG DRLTRAAKVL EOLTGOTPVF
SKARYTVRSF GIRRNEKIAV HCTVRGAKAE EILEKGLKVR EYELRKNNFS
DTGNFGFGIO EHIDLGIKYD PSIGIYGLDF YVVLGRPGFS IADKKRRTGC
IGAKHRISKE EAMRWFQQKY DGIILPGK

5. P62301 (100%), 17,223.3 Da
RS13_MOUSE 40S ribosomal protein S13 OS=Mus musculus GN=Rps13 PE=2 SV=2
3 exclusive unique peptides, 3 exclusive unique spectra, 3 total spectra, 30/151 amino acids (20% coverage)

MGRMHAPGKG LSOSALPYRR SVPTWLKLTS DDVKEOIYKL AKKGLTPSOI
GVILRDSHGV AOVRFVTGNK ILRILKSKGL APDLPEDLYH LIKKAVAVRK
HLERNRKDKD AKFRLILIES RIHRLARYYK TKRVLPPNWK YESSTASALV
A

6. P62911 (100%), 15,860.4 Da
RL32_MOUSE 60S ribosomal protein L32 OS=Mus musculus GN=Rpl32 PE=2 SV=2
2 exclusive unique peptides, 2 exclusive unique spectra, 2 total spectra, 27/135 amino acids (20% coverage)

MAALRPLVKP KIVKKRTKKF IRHOSDRYVK IKRNWRKPRG IDNRVRRRFK
GOILMPNIGY GSNKKTKHML PSGFRKFLVH NVKELEVLLM CNKSYCAEIA
HNVSSKNRKA IVERAAQLAI RVTNPNARLR SEENE

7. B1ARA3 (100%), 12,217.1 Da
B1ARA3_MOUSE 60S ribosomal protein L26 (Fragment) OS=Mus musculus GN=Rpl26 PE=2 SV=1
3 exclusive unique peptides, 3 exclusive unique spectra, 3 total spectra, 18/103 amino acids (17% coverage)

MKFNPFVTSD RSKNRKRHFN APSHIRRKIM SSPLSKELRQ KYNVRSMPIR
KDDEVQVVRG HYKGQQIGKV VQVYRKKYVI YIERVQREKA NGTTVHVGIH
PSK

8. P14115 (100%), 16,605.4 Da
RL27A_MOUSE 60S ribosomal protein L27a OS=Mus musculus GN=Rpl27a PE=2 SV=5
2 exclusive unique peptides, 2 exclusive unique spectra, 2 total spectra, 23/148 amino acids (16% coverage)

MPSRLRKTRK LRGHVSHGHG RIGKHRKHPG GRGNAGGMHH HRINFDKYHP
GYFGKVGMRH YHLKRNOSFC PTVNLDKLWT LVSEOTRVNA AKNKTGVAPI
IDVVRSGYYK VLGKGKLPKQ PVIVKAKFFS RRAEEKIKGV GGACVLVA

9. P62852 (100%), 13,743.0 Da
RS25_MOUSE 40S ribosomal protein S25 OS=Mus musculus GN=Rps25 PE=2 SV=1
2 exclusive unique peptides, 2 exclusive unique spectra, 2 total spectra, 18/125 amino acids (14% coverage)

MPPKDDKKKK DAGKSAKKDK DPVNKSGGKA KKKKWSKGKV RDKLNNLVLF
DKATYDKLCK EVPNYKLITP AVVSERLKIR GSLARAALQE LLSKGLIKLV
SKHRAQVIYT RNTKGGDAPA AGEDA

10. P63325 (100%), 18,916.3 Da
RS10_MOUSE 40S ribosomal protein S10 OS=Mus musculus GN=Rps10 PE=1 SV=1
2 exclusive unique peptides, 2 exclusive unique spectra, 2 total spectra, 23/165 amino acids (14% coverage)

MLMPKKNRIA IYELLFKEGV MVAKKDVHMP KHPELADKNV PNLHVMKAMO
SLKSRGYVKE OFAWRHFYWY LTNEGIOYLR DYLHLPPEIV PATLRRSRPE
TGRPRPKGPE GERPARFTRG EADRDTYRRS AVPPGADKKA EAGAGSATEF
QFRGGFGRGR GQPPQ

11. P62281 (100%), 18,431.3 Da
RS11_MOUSE 40S ribosomal protein S11 OS=Mus musculus GN=Rps11 PE=2 SV=3
3 exclusive unique peptides, 3 exclusive unique spectra, 3 total spectra, 19/158 amino acids (12% coverage)

MADIOTERAY OKOPTIFONK KRVLLGETGK EKLPRYYKNI GLGFKTPKEA
IEGTYIDKKC PFTGNVSIRG RILSGVVTKM KMORTIVIRR DYLHYIRKYN
RFEKRHKNMS VHLSPCFRDV QIGDIVTVGE CRPLSKTVRF NVLKVTKAAG
TKKQFQKF

Figure O

1. **P62908 (100%), 26,674.5 Da**
RS3_MOUSE 40S ribosomal protein S3 OS=Mus musculus GN=Rps3 PE=1 SV=1
13 exclusive unique peptides, 13 exclusive unique spectra, 22 total spectra, 136/243 amino acids (56% coverag

```
MAVQISKKRK FVADGIFKAE LNEFLTRELA EDGYSGVEVR VTPTRTEIII
LATRTQNVLG EKGRRIRELT AVVQKRFGFP EGSVELYAEK VATRGLCAIA
QAESLRYKLL GGLAVRRACY GVLRFIMESG AKGCEVVVSG KLRGQRAKSM
KFVDGLMIHS GDPVNYYVDT AVRHVLLRQG VLGIKVKIML PWDPSGKIGP
KKPLPDHVSI VEPKDEILPT TPISEQKGGK PEPPAMPQPV PTA
```

2. **P97351 (100%), 29,885.3 Da**
RS3A_MOUSE 40S ribosomal protein S3a OS=Mus musculus GN=Rps3a PE=1 SV=3
8 exclusive unique peptides, 8 exclusive unique spectra, 11 total spectra, 90/264 amino acids (34% coverage)

```
MAVGKNKRLT KGGKKGAKKK VVDPFSKKDW YDVKAPAMFN IRNIGKTLVT
RTQGTKIASD GLKGRVFEVS LADLQNDEVA FRKFKLITED VQGKNCLTNF
HGMDLTRDKM CSMVKKWQTM IEAHVDVKTT DGYLLRLFCV GFTKKRNNQI
RKTSYAQHQQ VRQIRKKMME IMTREVQTND LKEVVNKLIP DSIGKDIEKA
CQSIYPLHDV FVRKVKMLKK PKFELGKLME LHGEGGSSGK AAGDETGAKV
ERADGYEPPV QESV
```

3. **P62242 (100%), 24,206.4 Da**
RS8_MOUSE 40S ribosomal protein S8 OS=Mus musculus GN=Rps8 PE=2 SV=2
5 exclusive unique peptides, 6 exclusive unique spectra, 9 total spectra, 60/208 amino acids (29% coverage)

```
MGISRDNWHK RRKTGGKRKP YHKKRKYELG RPAANTKIGP RRIHTVRVRG
GNKKYRALRL DVGNFSWGSE CCTRKTRIID VVYNASNNEL VRTKTLVKNC
IVLIDSTPYR QWYESHYALP LGRKKGAKLT PEEEEILNKK RSKKIQKKYD
ERKKNAKISS LLEEQFQQGK LLACIASRPG QCGRADGYVL EGKELEFYLR
KIKARKGK
```

4. **P62702 (100%), 29,599.3 Da**
RS4X_MOUSE 40S ribosomal protein S4, X isoform OS=Mus musculus GN=Rps4x PE=2 SV=2
5 exclusive unique peptides, 7 exclusive unique spectra, 10 total spectra, 66/263 amino acids (25% coverage)

```
MARGPKKHLK RVAAPKHWML DKLTGVFAPR PSTGPHKLRE CLPLIIFLRN
RLKYALTGDE VKKICMQRFI KIDGKVRTDI TYPAGFMDVI SIDKTGENFR
LIYDTKGRFA VHRITPEEAK YKLCKVRKIF VGTKGIPHLV THDARTIRYP
DPLIKVNDTI QIDLETGKIT DFIKFDTGNL CMVTGGANLG RIGVITNRER
HPGSFDVVHV KDANGNSFAT RLSNIFVIGK GNKPWISLPR GKGIRLTIAE
ERDKRLAAKQ SSG
```

5. **P62754 (100%), 28,681.7 Da**
RS6_MOUSE 40S ribosomal protein S6 OS=Mus musculus GN=Rps6 PE=1 SV=1
4 exclusive unique peptides, 4 exclusive unique spectra, 5 total spectra, 49/249 amino acids (20% coverage)

```
MKLNISFPAT GCQKLIEVDD ERKLRTFYEK RMATEVAADA LGEEWKGYVV
RISGGNDKQG FPMKQGVLTH GRVRLLLSKG HSCYRPRRTG ERKRKSVRGC
IVDANLSVLN LVIVKKGEKD IPGLTDTTVP RRLGPKRASR IRKLFNLSKE
DDVRQYVVRK PLNKEGKKPR TKAPKIQRLV TPRVLQHKRR RIALKKQRTK
KNKEEAAEYA KLLAKRMKEA KEKRQEQIAK RRRLSSLRAS TSKSESSQK
```

6. **P47963 (100%), 24,306.4 Da**
RL13_MOUSE 60S ribosomal protein L13 OS=Mus musculus GN=Rpl13 PE=2 SV=3
4 exclusive unique peptides, 4 exclusive unique spectra, 4 total spectra, 42/211 amino acids (20% coverage)

```
MAPSRNGMIL KPHFHKDWQQ RVDTWFNQPA RKIRRRKARQ AKARRIAPRP
ASGPIRPIVR CPTVRYHTKV RAGRGFSLEE LRVAGIHKKV ARTIGISVDP
RRRNKSTESL QANVQRLKEY RSKLILFPRK PSAPKKGDSS AEELKLATQL
TGPVMPIRNV YKKEKARVIT EEEKNFKAFA SLRMARANAR LFGIRAKRAK
EAAEQDVEKK K
```

7. **Q9CR57 (100%), 23,564.9 Da**
RL14_MOUSE 60S ribosomal protein L14 OS=Mus musculus GN=Rpl14 PE=2 SV=3
3 exclusive unique peptides, 3 exclusive unique spectra, 4 total spectra, 37/217 amino acids (17% coverage)

```
MVFRRYVEVG RVAYISFGPH AGKLVAIVDV IDQNRALVDG PCTRVRRQAM
PFKCMQLTDF ILKFPHSARQ KYVRKAWEKA DINTKWAATR WAKKIDARER
KAKMTDFDRF KVMKAKKMRN RIIKTEVKKL QRAAILKASP KKAAVAKAAI
AAAAAAAAAK AKVPAKKATG PGKKAAGQKA PAQKAAGQKA APPAKGQKGQ
KTPAQKAPAP KAAGKKA
```

8. **P25444 (100%), 31,232.2 Da**
RS2_MOUSE 40S ribosomal protein S2 OS=Mus musculus GN=Rps2 PE=2 SV=3
4 exclusive unique peptides, 4 exclusive unique spectra, 6 total spectra, 39/293 amino acids (13% coverage)

```
MADDAGAAGG PGGPGGPGLG GRGGFRGGFG SGLRGRGRGR GRGRGRGRGA
RGGKAEDKEW IPVTKLGRLV KDMKIKSLEE IYLFSLPIKE SEIIDFFLGA
SLKDEVLKIM PVQKQTRAGQ RTRFKAFVAI GDYNGHVGLG VKCSKEVATA
IRGAIILAKL SIVPVRRGYW GNKIGKPHTV PCKVTGRCGS VLVRLIPAPR
GTGIVSAPVP KKLLMMAGID DCYTSARGCT ATLGNFAKAT FDAISKTYSY
LTPDLWKETV FTKSPYQEFT DHLVKTHTRV SVQRTQAPAV ATT
```

9. **Q5XJF6 (100%), 24,832.0 Da**
Q5XJF6_MOUSE Ribosomal protein OS=Mus musculus GN=Rpl10a PE=2 SV=1
2 exclusive unique peptides, 2 exclusive unique spectra, 2 total spectra, 20/217 amino acids (9% coverage)

```
MSSKVSRDTL YEAVREVLHG NQRKRRKFLE TVELQISLKN YDPQKDKRFS
GTVRLKSTPR PKFSVCVLGD QQHCDEAKAV DIPHMDIEAL KKLNKNKKLV
KKLAKKYDAF LASESLIKQI PRILGPGLNK AGKFPSLLTH NENMVAKVDE
VKSTIKFQMK KVLCLAVAVG HVKMTDDELV YNIHLAVNFL VSLLKKNWQN
VRALYIKSTM GKPQRLY
```

10. **P62918 (100%), 28,024.8 Da**
RL8_MOUSE 60S ribosomal protein L8 OS=Mus musculus GN=Rpl8 PE=2 SV=2
2 exclusive unique peptides, 2 exclusive unique spectra, 3 total spectra, 18/257 amino acids (7% coverage)

```
MGRVIRGQRK GAGSVFRAHV KHRKGAARLR AVDFAERHGY IKGIVKDIIH
DPGRGAPLAK VVFRDPYRFK KRTELFIAAE GIHTGQFVYC GKKAQLNIGN
VLPVGTMPEG TIVCCLEEKP GDRGKLARAS GNYATVISHN PETKKTRVKL
PSGSKKVISS ANRAVVGVVA GGGRIDKPIL KAGRAYHKYK AKRNCWPRVR
GVAMNPVEHP FGGGNHQHIG KPSTIRRDAP AGRKVGLIAA RRTGRLRGTK
TVQEKEN
```

Figure P

1. P63276 (100%), 15,524.5 Da
RS17_MOUSE 40S ribosomal protein S17 OS=Mus musculus GN=Rps17 PE=1 SV=2
5 exclusive unique peptides, 5 exclusive unique spectra, 5 total spectra, 53/135 amino acids (39% coverage)

MGRVRTKTVK KAARVIIEKY YTRLGNDFHT NKRVCEEIAI IPSKKLRNKI
AGYVTHLMKR IQRGPVRGIS IKLQEEERER RDNYVPEVSA LDQEIIEVDP
DTKEMLKLLD FGSLSNLQVT QPTVGMNFKT PRGAV

2. P62751 (100%), 17,696.2 Da
RL23A_MOUSE 60S ribosomal protein L23a OS=Mus musculus GN=Rpl23a PE=1 SV=1
5 exclusive unique peptides, 5 exclusive unique spectra, 5 total spectra, 56/156 amino acids (36% coverage)

MAPKAKKEAP APPKAEAKAK ALKAKKAVLK GVHSHKKKKI RTSPTFRRPK
TLRLRRQPKY PRKSAPRRNK LDHYAIIKFP LTTESAMKKI EDNNTLVFIV
DVKANKHQIK QAVKKLYDID VAKVNTLIRP DGEKKAYVRL APDYDALDVA
NKIGII

3. P35979 (100%), 17,805.0 Da
RL12_MOUSE 60S ribosomal protein L12 OS=Mus musculus GN=Rpl12 PE=1 SV=2
4 exclusive unique peptides, 4 exclusive unique spectra, 7 total spectra, 56/165 amino acids (34% coverage)

MPPKFDPNEV KVVYLRCTGG EVGATSALAP KIGPLGLSPK KVGDDIAKAT
GDWKGLRITV KLTIQNRQAQ IEVVPSASAL IIKALKEPPR DRKKQKNIKH
SGNITFDEIV NIARQMRHRS LARELSGTIK EILGTAQSVG CNVDGRHPHD
IIDDINSGAV ECPAS

4. P62301 (100%), 17,223.3 Da
RS13_MOUSE 40S ribosomal protein S13 OS=Mus musculus GN=Rps13 PE=2 SV=2
5 exclusive unique peptides, 5 exclusive unique spectra, 6 total spectra, 51/151 amino acids (34% coverage)

MGRMHAPGKG LSQSALPYRR SVPTWLKLTS DDVKEQIYKL AKKGLTPSQI
GVILRDSHGV AQVRFVTGNK ILRILKSKGL APDLPEDLYH LIKKAVAVRK
HLERNRKDKD AKFRLILIES RIHRLARYYK TKRVLPPNWK YESSTASALV
A

5. Q9CXW4 (100%), 20,253.2 Da
RL11_MOUSE 60S ribosomal protein L11 OS=Mus musculus GN=Rpl11 PE=1 SV=4
5 exclusive unique peptides, 5 exclusive unique spectra, 5 total spectra, 51/178 amino acids (29% coverage)

MAQDQGEKEN PMRELRIRKL CLNICVGESG DRLTRAAKVL EQLTGQTPVF
SKARYTVRSF GIRRNEKIAV HCTVRGAKAE EILEKGLKVR EYELRKNNFS
DTGNFGFGIQ EHIDLGIKYD PSIGIYGLDF YVVLGRPGFS IADKKRRTGC
IGAKHRISKE EAMRWFQQKY DGIILPGK

6. P62281 (100%), 18,431.3 Da
RS11_MOUSE 40S ribosomal protein S11 OS=Mus musculus GN=Rps11 PE=2 SV=3
4 exclusive unique peptides, 4 exclusive unique spectra, 4 total spectra, 48/158 amino acids (30% coverage)

MADIQTERAY QKQPTIFQNK KRVLLGETGK EKLPRYYKNI GLGFKTPKEA
IEGTYIDKKC PFTGNVSIRG RILSGVVTKM KMQRTIVIRR DYLHYIRKYN
RFEKRHKNMS VHLSPCFRDV QIGDIVTVGE CRPLSKTVRF NVLKVTKAAG
TKKQFQKF

7. Q6ZWN5 (100%), 22,592.5 Da
RS9_MOUSE 40S ribosomal protein S9 OS=Mus musculus GN=Rps9 PE=2 SV=3
8 exclusive unique peptides, 8 exclusive unique spectra, 9 total spectra, 54/194 amino acids (28% coverage)

MPVARSWVCR KTYVTPRRPF EKSRLDQELK LIGEYGLRNK REVWRVKFTL
AKIRKAAREL LTLDEKDPRR LFEGNALLRR LVRIGVLDEG KMKLDYILGL
KIEDFLERRL QTQVFKLGLA KSIHHARVLI RQRHIRVRKQ VVNIPSFIVR
LDSQKHIDFS LRSPYGGGRP GRVKRKNAKK GQGGAGAGDD EEED

8. P62911 (100%), 15,860.4 Da
RL32_MOUSE 60S ribosomal protein L32 OS=Mus musculus GN=Rpl32 PE=2 SV=2
3 exclusive unique peptides, 3 exclusive unique spectra, 3 total spectra, 37/135 amino acids (27% coverage)

MAALRPLVKP KIVKKRTKKF IRHQSDRYVK IKRNWRKPRG IDNRVRRRFK
GQILMPNIGY GSNKKTKHML PSGFRKFLVH NVKELEVLLM CNKSYCAEIA
HNVSSKNRKA IVERAAQLAI RVTNPNARLR SEENE

9. P62900 (100%), 14,463.2 Da
RL31_MOUSE 60S ribosomal protein L31 OS=Mus musculus GN=Rpl31 PE=2 SV=1
3 exclusive unique peptides, 3 exclusive unique spectra, 6 total spectra, 33/125 amino acids (26% coverage)

MAPAKKGGEK KKGRSAINEV VTREYTINIH KRIHGVGFKK RAPRALKEIR
KFAMKEMGTP DVRIDTRLNK AVWAKGIRNV PYRIRVRLSR KRNEDEDSPN
KLYTLVTYVP VTTFKNLQTV NVDEN

10. P62849 (100%), 15,423.8 Da
RS24_MOUSE 40S ribosomal protein S24 OS=Mus musculus GN=Rps24 PE=2 SV=1
3 exclusive unique peptides, 3 exclusive unique spectra, 4 total spectra, 35/133 amino acids (26% coverage)

MNDTVTIRTR KFMTNRLLQR KQMVIDVLHP GKATVPKTEI REKLAKMYKT
TPDVIFVFGF RTHFGGGKTT GFGMIYDSLD YAKKNEPKHR LARHGLYEKK
KTSRKQRKER KNRMKKVRGT AKANVGAGKK PKE

11. B1ARA3 (100%), 12,217.1 Da
B1ARA3_MOUSE 60S ribosomal protein L26 (Fragment) OS=Mus musculus GN=Rpl26 PE=2 SV=1
4 exclusive unique peptides, 4 exclusive unique spectra, 8 total spectra, 26/103 amino acids (25% coverage)

MKFNPFVTSD RSKNRKRHFN APSHIRRKIM SSPLSKELRQ KYNVRSMPIR
KDDEVQVVRG HYKGQQIGKV VQVYRKKYVI YIERVQREKA NGTTVHVGIH
PSK

12. P62852 (100%), 13,743.0 Da
RS25_MOUSE 40S ribosomal protein S25 OS=Mus musculus GN=Rps25 PE=2 SV=1
4 exclusive unique peptides, 4 exclusive unique spectra, 4 total spectra, 27/125 amino acids (22% coverage)

MPPKDDKKKK DAGKSAKKDK DPVNKSGGKA KKKKWSKGKV RDKLNNLVLF
DKATYDKLCK EVPNYKLITP AVVSERLKIR GSLARAALQE LLSKGLIKLV
SKHRAQVIYT RNTKGGDAPA AGEDA

13. P62717 (100%), 20,732.6 Da
RL18A_MOUSE 60S ribosomal protein L18a OS=Mus musculus GN=Rpl18a PE=2 SV=1
3 exclusive unique peptides, 3 exclusive unique spectra, 4 total spectra, 35/176 amino acids (20% coverage)

MKASGTLREY KVVGRCLPTP KCHTPPLYRM RIFAPNHVVA KSRFWYFVSQ
LKKMKKSSGE IVYCGQVFEK SPLRVKNFGI WLRYDSRSGT HNMYREYRDL
TTAGAVTQCY RDMGARHRAR AHSIQIMKVE EIAAGKCRRP AVKQFHDSKI
KFPLPHRVLR RQHKPRFTTK RPNTFF

14. P47963 (100%), 24,306.4 Da
RL13_MOUSE 60S ribosomal protein L13 OS=Mus musculus GN=Rpl13 PE=2 SV=3
4 exclusive unique peptides, 4 exclusive unique spectra, 4 total spectra, 42/211 amino acids (20% coverage)

MAPSRNGMIL KPHFHKDWQQ RVDTWFNQPA RKIRRRKARQ AKARRIAPRP
ASGPIRPIVR CPTVRYHTKV RAGRGFSLEE LRVAGIHKKV ARTIGISVDP
RRRNKSTESL QANVQRLKEY RSKLILFPRK PSAPKKGDSS AEELKLATQL
TGPVMPIRNV YKKEKARVIT EEEKNFKAFA SLRMARANAR LFGIRAKRAK
EAAEQDVEKK K

15. G3UZJ6 (100%), 14,630.0 Da
G3UZJ6_MOUSE 60S ribosomal protein L18 (Fragment) OS=Mus musculus GN=Rpl18 PE=2 SV=1
2 exclusive unique peptides, 2 exclusive unique spectra, 3 total spectra, 24/126 amino acids (19% coverage)

MVVHLPQGVD IRHNKDRKVR RKEPKSQDIY LRLLVKLYRF LARRTNSTFN
QVVLKRLFMS RTNRPPLSLS RMIRKMKLPG RENKTAVVVG TVTDDVRILE
VPKLKVCALR VSSRARSRIL KAGGKI

16. Q91V55 (100%), 22,877.0 Da
Q91V55_MOUSE 40S ribosomal protein S5 OS=Mus musculus GN=Rps5 PE=2 SV=1
3 exclusive unique peptides, 3 exclusive unique spectra, 3 total spectra, 33/204 amino acids (16% coverage)

MTEWEAATPA VAETPDIKLF GKWSTDDVQI NDISLQDYIA VKEKYAKYLP
HSAGRYAAKR FRKAQCPIVE RLTNSMMMHG RNNGKKLMTV RIVKHAFEII
HLLTGENPLQ VLVNAIINSG PREDSTRIGR AGTVRRQAVD VSPLRRVNQA
IWLLCTGARE AAFRNIKTIA ECLADELINA AKGSSNSYAI KKKDELERVA
KSNR

17. P41105 (100%), 15,733.7 Da
RL28_MOUSE 60S ribosomal protein L28 OS=Mus musculus GN=Rpl28 PE=1 SV=2
2 exclusive unique peptides, 2 exclusive unique spectra, 4 total spectra, 22/137 amino acids (16% coverage)

MSAHLQWMVV RNCSSFLIKR NKQTYSTEPN NLKARNSFRY NGLIHRKTVG
VEPAADGKGV VVVMKRRSGQ RKPATSYVRT TINKNARATL SSIRHMIRKN
KYRPDLRMAA IRRASAILRS QKPVVVKRKR TRPTKSS

18. P62264 (100%), 16,272.9 Da
RS14_MOUSE 40S ribosomal protein S14 OS=Mus musculus GN=Rps14 PE=2 SV=3
2 exclusive unique peptides, 2 exclusive unique spectra, 8 total spectra, 24/151 amino acids (16% coverage)

MAPRKGKEKK EEQVISLGPQ VAEGENVFGV CHIFASFNDT FVHVTDLSGK
ETICRVTGGM KVKADRDESS PYAAMLAAQD VAQRCKELGI TALHIKLRAT
GGNRTKTPGP GAQSALRALA RSGMKIGRIE DVTPIPSDST RRKGGRRGRR
L

19. P63325 (100%), 18,916.3 Da
RS10_MOUSE 40S ribosomal protein S10 OS=Mus musculus GN=Rps10 PE=1 SV=1
2 exclusive unique peptides, 2 exclusive unique spectra, 2 total spectra, 23/165 amino acids (14% coverage)

MLMPKKNRIA IYELLFKEGV MVAKKDVHMP KHPELADKNV PNLHVMKAMQ
SLKSRGYVKE QFAWRHFYWY LTNEGIQYLR DYLHLPPEIV PATLRRSRPE
TGRPRPKGPE GERPARFTRG EADRDTYRRS AVPPGADKKA EAGAGSATEF
QFRGGFGRGR GQPPQ

20. P14131 (100%), 16,445.9 Da
RS16_MOUSE 40S ribosomal protein S16 OS=Mus musculus GN=Rps16 PE=2 SV=4
2 exclusive unique peptides, 2 exclusive unique spectra, 3 total spectra, 21/146 amino acids (14% coverage)

MPSKGPLQSV QVFGRKKTAT AVAHCKRGNG LIKVNGRPLE MIEPRTLQYK
LLEPVLLLGK ERFAGVDIRV RVKGGGHVAQ IYAIRQSISK ALVAYYQKYV
DEASKKEIKD ILIQYDRTLL VADPRRCESK KFGGPGARAR YQKSYR

21. P62843 (99%), 17,040.8 Da
RS15_MOUSE 40S ribosomal protein S15 OS=Mus musculus GN=Rps15 PE=2 SV=2
1 exclusive unique peptides, 1 exclusive unique spectra, 1 total spectra, 19/145 amino acids (13% coverage)

MAEVEQKKKR TFRKFTYRGV DLDQLLDMSY EQLMQLYSAR QRRRLNRGLR
RKQHSLLKRL RKAKKEAPPM EKPEVVKTHL RDMIILPEMV GSMVGVYNGK
TFNQVEIKPE MIGHYLGEFS ITYKPVKHGR PGIGATHSSR FIPLK

22. Q8BP67 (100%), 17,779.5 Da
RL24_MOUSE 60S ribosomal protein L24 OS=Mus musculus GN=Rpl24 PE=2 SV=2
2 exclusive unique peptides, 3 exclusive unique spectra, 3 total spectra, 21/157 amino acids (13% coverage)

MKVELCSFSG YKIYPGHGRR YARTDGKVFQ FLNAKCESAF LSKRNPRQIN
WTVLYRRKHK KGQSEEIQKK RTRRAVKFQR AITGASLADI MAKRNQKPEV
RKAQREQAIR AAKEAKKAKQ ASKKTAMAAA KAPTKAAPKQ KIVKPVKVSA
PRVGGKR

23. Q6ZWZ7 (100%), 21,397.5 Da
Q6ZWZ7_MOUSE 60S ribosomal protein L17 OS=Mus musculus GN=Rpl17 PE=2 SV=1
2 exclusive unique peptides, 2 exclusive unique spectra, 5 total spectra, 19/184 amino acids (10% coverage)

MVRYSLDPEN PTKSCKSRGS NLRVHFKNTR ETAQAIKGMH IRKATKYLKD
VTLKKQCVPF RRYNGGVGRC AQAKQWGWTQ GRWPKKSAEF LLHMLKNAES
NAELKGLDVD SLVIEHIQVN KAPKMRRRTY RAHGRINPYM SSPCHIEMIL
TEKEQIVPKP EEEVAQKKKI SQKKLKKQKL MARE

24. P62267 (99%), 15,807.7 Da
RS23_MOUSE 40S ribosomal protein S23 OS=Mus musculus GN=Rps23 PE=2 SV=3
1 exclusive unique peptides, 1 exclusive unique spectra, 1 total spectra, 11/143 amino acids (8% coverage)

MGKCRGLRTA RKLRSHRRDQ KWHDKQYKKA HLGTALKANP FGGASHAKGI
VLEKVGVEAK QPNSAIRKCV RVQLIKNGKK ITAFVPNDGC LNFIEENDEV
LVAGFGRKGH AVGDIPGVRF KVVKVANVSL LALYKGKKER PRS

Figure legends

Figure A. Quantification of RP transcript relative expression in murine HBs. (A) Differential abundance of RP transcripts between WT hepatocytes and WT HBs. Data are derived from the RNA-seq results shown in Fig 1A. Actual fractional abundance is shown with the total adding to 100%. Asterisks indicate those transcripts in HBs whose abundance relative to that of the same transcript in hepatocytes differs (q-value <0.05 after FDR-adjustment). Error bars indicate one standard deviation. (B) The same representation as shown in (A) for KO hepatocytes and HBs.

Figure B. Quantification of RP transcript relative expression over the course of HCC induction, regression and recurrence. Data are presented as described in Figure A. Each time point is compared to normal liver results. (Asterisks indicate p<0.05 after FDR-adjustment).

Figure C. Additional immuno-blots of RPs from representative tissue samples over the course of HCC induction, regression and recurrence. Blots were performed as described for Fig 1F. Three additional groups are shown here.

Figure D. 3D Area Plot depicting RP transcript deregulation in human HCCs. The data shown include the matched HCC samples depicted in Fig 3A as well as 323 additional unmatched samples. F-tests were performed comparing variance in relative expression for each RP transcript across the normal matched liver samples and the HCC tumors in order to determine if the variability in RP transcript expression was significantly different across tumors compared

to normal liver. F-tests were significant for 69 of 77 RPs after FDR adjustment. The RP genes Rps26, Rpl9, Rps27, Rps28, and Rpl21, with the lowest expression and greatest variability (without significant F-tests), were excluded from the graph in order to appreciate differences in the other transcripts.

Figure E. 3D Area Plot depicting RP transcript deregulation in human CRCs. Data are presented as described in Figure D and include the matched tumor samples shown in Fig 3B. F-tests were significant in 48 of 77 RPs, and the same five transcripts were excluded from the graph.

Figure F. 3D Area Plot depicting RP transcript deregulation in human breast cancers. Data are presented as described in Figure D and include the matched tumor samples shown in Fig 3C. F-tests were significant in 71 of 77 RPs, and the same five transcripts were excluded from the graph.

Figure G. 3D Area Plot depicting RP transcript deregulation in human prostate cancers. Data are presented as described in Figure D and include the matched tumor samples shown in Fig 3D. F-tests were significant in 23 of 77 RPs, and the same five transcripts were excluded from the graph.

Figure H. The relationship between ribosomal stress and the p19^{ARF}/Mdm2/ p53 pathway. The complex and highly regulated process of ribosomal biogenesis [21, 22, 26] can be disrupted

by RP haplo-insufficiency, leading to ribosomal stress [1, 2, 4]. In response, p19*ARF* is induced and inhibits further ribosomal biogenesis by blocking the nucleolar export of 40S and 60S ribosomal subunits [3, 8, 55]. Independently, p19*ARF* can inhibit Mdm2 and thus prevent the latter protein's binding to and promoting the ubiquitin-mediated degradation of p53 [30]. A subset of free RPs can also bind the p53-recognition domain of Mdm2 and prevent or disrupt the Mdm2-p53 interaction [32]. Additionally, at least one RP, namely RPS26, has recently been shown to interact directly with p53 and supplement its transcriptional activity [33].

Figure I. Immuno-blotting for p19ARF, Mdm2 and p53 in additional HCCs. Studies were performed as described in Fig 5B.

Figure J. Mdm2-interacting RPs identified in HCCs. (A) p53 and Mdm2 co-localize to HCC cytoplasm. A freshly collected HCC tumor was fractionated into cytoplasmic, nuclear and nucleolar compartments. Each fraction was tested for the protein markers localizing to these compartments (GAPDH, histone H3 and fibrillarin, respectively) and in parallel for p53, p19ARF and Mdm2. Varying amounts of lysate and exposure times were required to compensate for differential expression of the proteins. (B) Liver and HCC cytoplasmic fractions were immuno-precipitated with control IgG or anti-Mdm2 IgG. Precipitates were resolved by SDS-PAGE and silver stained. Bracketed regions were excised from HCC MDM2-IP lane and subjected to tryptic digestion and mass spectrometry. (C) A Venn diagram showing the comparison of RPs detected in MDM2-IPs between liver, HBs and HCCs. Eight Mdm2-interacting RPs were common with HB while an additional nine were unique to HCCs shown also in Table E and Figures O and P.

Figure K. Mdm2-interacting RP peptides identified by in HB cytoplasmic lysates following anti-Mdm2 IP (~24-35 kDa range). HB cytoplasmic extracts were immuno-precipitated with agarose-linked anti-Mdm2 antibody. After release of the Mdm2-interacting proteins and resolution by SDS-PAGE, the silver-stained region of the gel corresponding to the ca. 24-35 kDa region of lane 4 (Fig 5E, red bracket) was excised and subjected to tryptic digestion and mass spectrometric analysis as described in Materials and methods. Peptides identified in the analysis are highlighted in yellow on their corresponding full-length RP. The coverage for each RP ranged from 10-64%. Each of the 12 RPs listed here is also listed in S2 Table.

Figure L. Mdm2-interacting RP peptides identified by MS in liver cytoplasmic lysates following anti-Mdm2 IP (~24-35 kDa range). An analysis identical to that described in S3 Table was performed the portion of the gel denoted by the red band depicted in Fig 5E. The coverage for each RP ranged from 7-39%. Each of the 7 RPs listed here is also listed in Table B.

Figure M. Mdm2-interacting RP peptides identified by MS in HB cytoplasmic lysates following anti-Mdm2 IP (~14-24 kDa range). Tryptic peptides corresponding to the indicated 17 RPs in the ~14-24 kDa range identified by mass spectrometry lane 4, Fig 5E (blue bracket) and listed in Table D. Each identified peptide is indicated by yellow highlighting and is mapped to its corresponding region in the sequence of the full-length RP. The coverage for each RP ranged from 13-59%.

Figure N. Mdm2-interacting RP peptides identified in liver cytoplasmic lysates following anti-Mdm2 IP (~14-24 kDa range). Tryptic peptides corresponding to the indicated 11 RPs in the ~14-24 kDa range identified by mass spectrometry (Fig 4E, lane 2, blue bracket) and listed in Table D are indicated by yellow highlighting and is mapped to its corresponding region in the sequence of the full-length RP. The coverage for each RP ranged from 12-38%.

Figure O. Mdm2-interacting RPs identified by MS in HCC cytoplasmic lysates following anti-MDM2-IP (~24-35 kDa range). HCC cytoplasmic extracts were immuno-precipitated with agarose-linked anti-Mdm2 antibody. After release of the Mdm2-interacting proteins and resolution by SDS-PAGE, the silver-stained region of the gel corresponding to the ca. 24-35 kDa region of HCC MDM2-IP lane (Figure J, red bracket) was excised and subjected to tryptic digestion and mass spectrometric analysis as described in Materials and methods. Peptides identified in the analysis are highlighted in yellow on their corresponding RP. The coverage for each RP ranged from 7-56%. Each of the 10 RPs listed here is also listed in Table E.

Figure P. Mdm2-interacting RP peptides identified by MS in HCC cytoplasmic lysates following anti-Mdm2 IP (~14-24 kDa range). Tryptic peptides corresponding to the indicated 24 RPs in the ~14-24 kDa range as identified by mass spectrometry from MDM2-IP lane, Figure J (blue bracket) are listed in Table E. Each identified peptide is indicated by yellow highlighting and is mapped to its corresponding region in the sequence of the full-length RP. The coverage for each RP ranged from 8-39%.

Tables

Table A

Antibody	Species	Vendor and catalog No.	Dilution used
RPS10	Rabbit	GeneTex (GTX101836)	1:1000
RPS19	Rabbit	GeneTex (GTX54725)	1:1000
RPS24	Rabbit	GeneTex (GTX47408)	1:1000
RPS26	Rabbit	Proteintech (14909-1-AP)	1:1000
RPS27	Goat	OriGene (TA302828)	1:1000
RPL5	Rabbit	GeneTex (GTX101821)	1:1000
RPL11	Rabbit	Abcam (79352)	1:1000
RPL26	Rabbit	Bethyl Laboratories (A300-685A-T)	1:1000
RPL30	Rabbit	GeneTex (GTX87885)	1:500
BCL-2	Rabbit	Cell Signaling (28700)	1:300
GAPDH	Mouse	Sigma-Aldrich (G8795)	1:20,000
HRP anti-mouse	Horse	Cell Signaling (7076)	1:10,000
HRP anti-rabbit	Goat	Cell Signaling (7074)	1:5000
Alexa Fluor 488 anti-rabbit	Goat	Thermo Fisher (A-11008)	1:1000
AC-Histone H3	Rabbit	Santa Cruz (SC8655-R)	1:500
Fibrillarin	Rabbit	Cell Signaling (2639)	1:1000
p19 ARF	Rabbit	Santa Cruz (SC22784)	1:500
MDM2	Mouse	Santa Cruz (SC965)	1:1000
P53	Mouse	Calbiochem(OP03)	1:1000
Mdm2 (for immunostain)	Rabbit	Abcam (Ab38618)	1:200
P53 (for immunostain)	Goat	Santa Cruz, (SC6243-G)	1:50
BAX	Rabbit	Cell signaling (2772)	1:1000
Caspase-2	Mouse	Santa Cruz (SC514472)	1:500

Table B

Gene	Cohorts	Average Expression Difference vs. Normal Tissues
Rpl36a	HCC, BC, CRC, PC	131.40%
Rpl28	HCC, BC, PC	40.24%
Rps21	HCC, BC, CRC, PC	33.41%
Rpl8	HCC, BC, CRC, PC	33.17%
Rpl30	HCC, BC, CRC	28.20%
Rps2	HCC, BC, CRC, PC	27.62%
Rpl39	HCC, CRC, PC	26.04%
Rpl36	BC, CRC, PC	21.94%
Rps19	HCC, BC, CRC, PC	21.94%
Rpl38	HCC, BC, CRC, PC	18.37%
Rplp0	HCC, BC, PC	12.76%
Rpl23	HCC, BC, CRC, PC	11.15%
Rps16	HCC, BC, PC	10.97%
Rpl23a	HCC, BC, PC	10.16%
Rps10	HCC, BC, PC	9.02%
Rpl37	HCC, BC, CRC, PC	8.95%
Rps7	HCC, CRC, PC	8.94%
Rps24	BC, CRC, PC	7.13%
Rpl35a	HCC, CRC, PC	4.30%
Rps4x	BC, CRC, PC	-4.03%
Rpl4	HCC, BC, PC	-6.45%
Rps12	BC, CRC, PC	-7.42%
Rps6	HCC, BC, PC	-9.45%
Rpl17	BC, CRC, PC	-9.99%
Rps14	BC, CRC, PC	-11.06%
Rps23	BC, CRC, PC	-11.19%
Rpl10a	BC, CRC, PC	-11.63%
Rps13	HCC, BC, CRC	-13.55%
Rpl3	HCC, BC, CRC, PC	-15.68%
Rps25	HCC, BC, CRC	-16.42%
Rpl15	HCC, BC, CRC, PC	-16.58%
Rpl22	HCC, BC, CRC, PC	-17.31%
Rps3a	HCC, BC, CRC, PC	-17.42%
Rpl5	BC, CRC, PC	-18.57%
Rpl11	HCC, BC, CRC	-18.72%
Rpl34	HCC, BC, CRC	-21.37%
Rpl26	HCC, BC, CRC	-23.71%

Table C

HB cytoplasmic extract		Normal liver cytoplasmic extract	
RPL7	31 kDa*	ND	
RPL8	28 kDa	RPL8	28 kDa
RPL10A	25 kDa*	ND	
RPL13	24 kDa	RPL13	24 kDa
RPL14	24 kDa*	ND	
RPS2	31 kDa*	ND	
RPS3	27 kDa	RPS3	27 kDa
RPS3A	30 kDa	RPS3A	30 kDa
RPS4X	30 kDa	RPS4X	30 kDa
RPS6	29 kDa	RPS6	29 kDa
RPS8	24 kDa	RPS8	24 kDa
RPSA	33 kDa*	ND	

*Detected only in HB
ND = not detected

Table D

HB cytoplasmic extract		Normal liver cytoplasmic extract	
RPL11	20 kDa	RPL11	20 kDa
RPL12	18 kDa	RPL12	18 kDa
RPL13	24 kDa*	ND	
RPL23A	18 kDa*	ND	
RPL24	18 kDa	RPL24	15 kDa
RPL26	17 kDa	RPL26	17 kDa
RPL27A	17 kDa	RPL27A	17 kDa
RPL28	16 kDa*	ND	
RPL32	16 kDa	RPL32	16 kDa
RPL35	15 kDa*	ND	
RPS10	19 kDa	RPS10	19 kDa
RPS11	18 kDa	RPS11	18 kDa
RPS13	17 kDa	RPS13	17 kDa
RPS15	17 kDa*	ND	
RPS17	16 kDa*	ND	
RPS24	15 kDa*	ND	
RPS25	14 kDa	RPS25	14 kDa
ND		RPS18	18 kDa^{+}

*Detected only in HB

$^{+}$Detected only in normal liver

ND = not detected

Table E

HCC-D (~24-35 kDa)		HCC-E (~14-24 kDa)	
		RPL13	24 kDa
		RPS9	23 kDa*
		RPL26	12 kDa (FRAGMENT)
		RPS14	16 kDa*
		RPL12	18 kDa
		RPS13	17 kDa
		RPL31	14 kDa*
RPS3	27 kDa	RPL23A	18 kDa*
RPS3A	30 kDa	RPS17	16 kDa*
RPS4X	30 kDa	RPL11	20 kDa
RPL13	24 kDa	RPS11	18 kDa
RPS8	24 kDa	RPS25	14 kDa*
RPS6	29 kDa	RPS24	15 kDa*
RPS2	31 kDa*	RPL18A	21 kDa*
RPL8	28 kDa	RPL28	16 kDa*
RPL14	24 kDa*	RPS5	23 kDa*
RPL10A	25 kDa*	RPL32	16 kDa
		RPL18	15 kDa (FRAGMENT)*
		RPL24	18 kDa
		RPS16	16 kDa*
		RPS10	19 kDa
		RPS23	16 kDa*
		RPS15	17 kDa*
		RPL17	21 kDa*

*Present only in HCC when compared to normal liver

Table F

Gene	HCC	CRC	BC	PC
RpsA		Q261R		
Rps2	V209A	Splice region	F84L	
Rps3	Q4R (2)	A71D, G15R		
Rps3a	D196Tfs*2, V22A			
Rps4x		Y54C, V207M	I102M, N224H	
Rps4y1	Y149Sfs*9			
Rps5			E98K, E3K, V113L, F20L, V11G, T126A	R130H
Rps6	L133P, K221Lfs*26		S236C, S139N, K93N, M1?	R232H
Rps7	I60V			
Rps8			C71W	
Rps9	R54Afs*8	R79W, R83H, I32I		R133P
Rps11		G53Afs*24		R139S, K32T
Rps12			E87K	
Rps13	P7S	I37M		
Rps15a		N91K		
Rps16		R62X, R69L	L47V	
Rps18		F9S, T145A	I117N	
Rps20		R79C		A16T
Rps23	V55Sfs*4	G33D, G78D, N39T, V85L		
Rps24	T38A			
Rps25			A87G	
Rps27	D6N		Splice region	
Rps27a	L56Cfs*16			K83del, Y106C
Rps29			S20F	
Fau	N132Y, V86del	Y112C		

Table G

Gene	HCC	CRC	BC	PC
Rpl3	R174H, G225D, A359S	K124T, T346A	V338G, V85G	K128del
Rpl4	Splice region	E13K, R97C	D179H	
Rpl5	D59E	K258N	N57Efs*12, A97G	N94D, G156del, A77T
Rpl6		F193C (2), T213I		T148N, N101S
Rpl7		R22Q	V4G	V14G, E188Q
Rpl7a	R196G, R59H, R89L			P58S
Rpl8	I158F		I112Qfs*38, A27V	R3H
Rpl9	R125M	I151T, R115Q, R89H, N42D	L176V	
Rpl10		L103S	I97M, I70L	A155T
Rpl10a	K98Q, R7G	P135Lfs*17, A166V		L138F, E88*, E178K
Rpl11	R146C, T148A, G86C	F166L		L15F, K8Rfs*26, Y131C, C72R
Rpl12			V42Lfs*15	R117W
Rpl13	R183C, Q111*			
Rpl13a			T132I, N65K, R37G, V203L	
Rpl15			R189Gfs*18, S187Ffs*29	
Rpl18	L27M		G118V	
Rpl18a	R116Gfs*12, Q144L, V62F	R43C	R83H, R95Q, R43Pfs*10, R166C	
Rpl19	R16C	A159V	R107K, E28K	K21del, R151H
Rpl21	I93L	F15L		
Rpl22	C25*	K89Nfs*3, K15Rfs*5	K15Rfs*5 (4)	
Rpl23	K75Rfs*31		E99Q	
Rpl23a	I76V			
Rpl24		P133H	R105Q	
Rpl26			R50Q	
Rpl27	R21H			
Rpl27a	K7R			
Rpl28	V78M	D105N, P53S		K65M
Rpl29				R44C
Rpl31		Splice region, E94V	R85H	
Rpl32		R27Q	F20L	
Rpl34				A16T
Rpl35		R84Q	K79N	
Rpl35a	Y14C			V33I
Rpl36			I81M	
Rpl36a	A60Gfs*2		R57Q	
Rpl37	N13S			A51G
Rpl37a			K62dup	
Rpl39		R21C		
Rpl41			R21T	
Rplp0	P272Lfs*63	V121A, F316del	A278G (2), A262T, E299K	
Rplp1				150V
Rplp2			E92Q	
Uba52	Q62*	M94T, L71S	H104Y, N90Y	

Table H

Gene Symbol	Number of Non-RP-Mutant Tumors with Mutation (Total = 192)	Number of RP-Mutant Tumors with Mutation (Total = 31)	P-value
TTN	62	22	4.55×10^{-15}
RYR2	13	17	2.14×10^{-9}
MUC16	23	15	1.59×10^{-7}
LRP1B	23	15	1.59×10^{-7}
FAT4	25	15	1.59×10^{-7}
NEB	5	14	1.14×10^{-6}
DNAH10	8	14	1.14×10^{-6}
CSMD2	10	14	1.14×10^{-6}
LRP2	21	14	1.14×10^{-6}
SYNE1	35	14	1.14×10^{-6}
USH2A	14	13	7.18×10^{-6}
CSMD3	15	13	7.18×10^{-6}
HMCN1	17	13	7.18×10^{-6}
PIK3CA	32	13	7.18×10^{-6}
ATR	5	12	4.02×10^{-5}
DNAH11	6	12	4.02×10^{-5}
DOCK2	9	12	4.02×10^{-5}
RYR3	11	12	4.02×10^{-5}
ACVR2A	13	12	4.02×10^{-5}
CSMD1	15	12	4.02×10^{-5}
FAT2	17	12	4.02×10^{-5}
DNAH5	22	12	4.02×10^{-5}
ARAP2	2	11	1.99×10^{-4}
CHD6	3	11	1.99×10^{-4}
PAPPA2	3	11	1.99×10^{-4}
PKHD1L1	4	11	1.99×10^{-4}
ZNF292	5	11	1.99×10^{-4}
KIAA1109	5	11	1.99×10^{-4}
FBN1	6	11	1.99×10^{-4}
LAMA2	6	11	1.99×10^{-4}
MAP1B	7	11	1.99×10^{-4}
DST	8	11	1.99×10^{-4}
DNAH8	8	11	1.99×10^{-4}
ANK3	8	11	1.99×10^{-4}
PCDH15	9	11	1.99×10^{-4}
PCLO	9	11	1.99×10^{-4}
PDZD2	9	11	1.99×10^{-4}
MYH11	10	11	1.99×10^{-4}
MACF1	11	11	1.99×10^{-4}
RYR1	13	11	1.99×10^{-4}
DMD	20	11	1.99×10^{-4}

Table legends.

Table A. Antibodies utilized in the current study.

Table B. Shared RP transcript deregulation across human cancers. Listed are RP transcripts which showed significantly different relative expression in tumors compared to normal tissues, as well as shared directionality in at least 2 of the 4 cancer cohorts examined. BC = breast cancer, HCC = hepatocellular carcinoma, CRC = colorectal carcinoma, PC = prostate cancer. For each RP transcript, relative percent expression was compared between tumor (all matched and unmatched samples) and average relative expression in normal tissue with a two-sided t-test ($P < 0.05$). All P-values were then adjusted based on a false-discovery rate of 5%. Percent difference in relative expression for a given transcript was then calculated by dividing the difference in average relative expression between HCC and normal tissue by the average relative expression in normal tissue. Transcripts were defined as having shared directionality when differences in relative percent expression were either all increased or decreased relative to normal tissues. Transcripts with significantly different relative percent expression in 2 or more cancers but without shared directionality were excluded.

Table C. Mdm2-interacting RPs identified in HB and normal liver cytoplasm (~24-35 kDa range). Note that all seven of the RPs associated with Mdm2 in normal liver cytoplasmic lysates were also identified in IPs from HB cytoplasmic lysates. See Figure K and Figure L for the identities of all detected peptides. Among the non-RPs identified by mass spectrometry in this analysis were five isoforms of the 14-3-3 family which have been previously identified as Mdm2 partners (56-60).

Table D. Mdm2-interacting RPs identified in HB and normal liver cytoplasmic lysates following anti-Mdm2 IP (~14-24 kDa range; blue bracket in Fig 5E). Note that 10 of 11 proteins identified as Mdm2 binding partners in normal liver were also identified in IPs from HBs, which contained seven additional RPs. See Figure M and Figure N for the exact mapping of each identified peptide to its corresponding RP.

Table E. Mdm2-interacting RPs identified in HCC cytoplasmic lysates following anti-Mdm2 IP. See Figure O and Figure P for the exact mapping of each identified peptide to its corresponding RP.

Table F. **Mutations in RP small subunit genes identified in four investigated TCGA cancer cohorts, designated with standard HGVS nomenclature.**

Table G. **Mutations in RP large subunit genes identified in four investigated TCGA cancer cohorts, designated with standard HGVS nomenclature.**

Table H. **Mutations associated with ribosomal protein mutations in human colorectal cancers.** The listed genes are more frequently co-mutated in tumors possessing a ribosomal protein mutation than would be expected by chance alone. P-values were calculated using cumulative binomial distributions and are significant after correction for false discovery.
